# The molecular landscape of sepsis severity in infants: enhanced coagulation, innate immunity, and T cell repression

**DOI:** 10.3389/fimmu.2024.1281111

**Published:** 2024-05-16

**Authors:** Susie Shih Yin Huang, Mohammed Toufiq, Pirooz Eghtesady, Nicholas Van Panhuys, Mathieu Garand

**Affiliations:** ^1^ Division of Pediatric Cardiothoracic Surgery, Department of Surgery, Washington University School of Medicine, St. Louis, MO, United States; ^2^ Department of Immunology, Sidra Medicine, Doha, Qatar

**Keywords:** biomarker, transcriptomic, infant sepsis, sepsis severity, blood, immune deconvolution

## Abstract

**Introduction:**

Sepsis remains a major cause of mortality and morbidity in infants. In recent years, several gene marker strategies for the early identification of sepsis have been proposed but only a few have been independently validated for adult cohorts and applicability to infant sepsis remains unclear. Biomarkers to assess disease severity and risks of shock also represent an important unmet need.

**Methods:**

To elucidate characteristics driving sepsis in infants, we assembled a multi-transcriptomic dataset from public microarray datasets originating from five independent studies pertaining to bacterial sepsis in infant < 6-months of age (total n=335). We utilized a COmbat co-normalization strategy to enable comparative evaluation across multiple studies while preserving the relationship between cases and controls.

**Results:**

We found good concordance with only two out of seven of the published adult sepsis gene signatures (accuracy > 80%), highlighting the narrow utility of adult-derived signatures for infant diagnosis. Pseudotime analysis of individual subjects’ gene expression profiles showed a continuum of molecular changes forming tight clusters concurrent with disease progression between healthy controls and septic shock cases. In depth gene expression analyses between bacteremia, septic shock, and healthy controls characterized lymphocyte activity, hemostatic processes, and heightened innate immunity during the molecular transition toward a state of shock.

**Discussion:**

Our analysis revealed the presence of multiple significant transcriptomic perturbations that occur during the progression to septic shock in infants that are characterized by late-stage induction of clotting factors, in parallel with a heightened innate immune response and a suppression of adaptive cell functionality.

## Introduction

Sepsis affects over 19 million patients annually, with global mortality rates at around 25-30% (1–3). Although palliative care and anti-microbial treatment have markedly improved sepsis management, mortality rates remains high due to disease heterogeneity, highly variable host characteristics, including cardiovascular and immunological comorbidities, and shortcomings in methods for early detection and diagnosis (4, 5). In young infants, the detection of sepsis is often compounded by other events, including systemic inflammatory response to trauma or surgery, and signs of infection can be rather subtle due to the immaturity of the immune system (6). A positive bacterial culture is the current definitive criterion used for diagnosis of infection, with downsides including lengthy culture times (24-48hrs) and incidents of false negative/positive results (7, 8). The prospect for significant improvement of patient outcomes through early detection of infections has motivated the investigation of alternate predictors or markers, including gene signatures (9).

Several gene signatures for sepsis development have been established from adult cohorts (10–16) while information from younger cohorts has been more difficult to obtain. Currently, there are only a handful of published transcriptomic signatures for neonates and infants with sepsis (11, 12, 17–19). Sweeney et al. (2018) validated a gene-based diagnostic signature in the neonate age group, achieving an accuracy of 0.9 for sepsis classification among three independent cohorts (11). However, the differentially modulated genes and the underlying biological perturbations responsible for inducing the genetic signature remain to be explored. The immunological cascades in infants are different than in more mature age groups (17, 18). Previous comparative models to distinguish viral and bacterial infections have determined the existence of differential immunological features between infants and older cohorts, highlighting the need for specific studies in infant sepsis (20). The period spanning from 0 to 6 months is recognized as a phase of heightened vulnerability to infections, characterized by a unique immune state that merits further investigation. This distinctive immunological landscape during early infancy underscores the importance of delving deeper into the intricacies of this critical developmental stage.

In order to accurately refine and apply predictive molecular signature(s) to infant sepsis, it is critical to better understand the biological processes and immune responses as they occur in infants. As such, we queried publicly available microarray studies which include infants with bacterial sepsis (henceforth referred to “bacteremia”), septic shock, and healthy controls to increase sample size and statistical power for detecting differences in their gene expression profiles. A total of five datasets, consisting of 335 subjects, formed the multi-transcriptome (henceforth referred to “merged dataset”). Successive normalization steps were employed to minimize inter-dataset variability and the impact of potential confounders. Several published sepsis gene marker signatures were then tested (11–16, 21). Here, we recapitulated some expected signatures, whilst the majority tended to be specific to the age of the derived cohort. Differentially expressed genes (DEGs) were generated between Bacteremia, Septic Shock, and Healthy Controls groups and examined for their putative roles by over-representation enrichment analyses (ORA). We also performed the analyses with computationally determined pseudotime clusters in a ‘side-by-side’ fashion. We show that molecular perturbations among infants with Bacteremia spread over a pseudotime continuum and largely fit into clusters resembling Healthy Controls, Septic Shock, or a transitory state. Importantly, the pseudotime clustering revealed a transition from adaptive immune cues, such as IFN-gamma production, towards greater activation of innate cells and pathways coincident with progression of disease severity.

## Materials and methods

### Study design and dataset selection

The study is a re-analysis of publicly available microarray datasets. In brief, searches for genome-expression studies of infant sepsis (up to 6 months of age) were conducted in PubMed, NCBI GEO, and EBI ArrayExpress. We also leveraged the availability of our recently published transcriptomic dataset collection on sepsis, called *SysInflam HuDB* (22), to identify the appropriate studies. The keywords “neonate* AND sepsis” as well as “infant* AND sepsis” were used to query the databases. Only datasets which included both infants with sepsis and a reference/control class, where ages of the individual subject were specified and gestational ages > 30 weeks were included.

### Sepsis definition

Categories of sepsis were determined from the associated clinical data of the original publications with the consensus that blood culture positivity is the established standard for the diagnosis; therefore, each case represents bacterial sepsis and is referred to as bacteremia in this study. The study-specific definitions are provided as **Supplementary Information**.

### Data processing and normalization

When available, raw data were downloaded and datasets were individually normalized using RMA normalization method (for Affymetrix microarray) or normal-exponential background correction followed by quantile normalization (for Illumina microarray) unless indicated (**Table 1**). GSE25504 is composed of three platforms. Data were then log_2_-transformed and the probes summarized to genes, using the mean if a gene was matched to multiple probes. The resulting median gene number per platform was 21,107 (18,947 to 22,296). Only genes that are present in all platforms were retained (8466 genes) for further analysis.

**Table 1:** Description of the datasets retrieved from public databases and study characteristics associated with each dataset.

GSE Datasets	Platform	Platform	Disease definition	Source of definition	further details	ICD-10	Total # of samples	Patient	Size (Patient)	Healthy Control	Size (Healthy Control)	Individual dataset normalization	Associated paper	Study type	Clinical setting of enrollment	Sample collected
GSE64456	GPL10558	Illumina HumanHT-12 V4.0 expression beadchip	Sepsis	Positive blood culture	Based on culture results, febrile infants were assigned either to with or without bacterial infection group (i.e., bacteremia from single pathogen, UTI, bacterial meningitis).	A41.89	46	Sepsis bacteremia	32	Healthy Control	14	Raw data obtained from GEO -> Normal-exponential background correction -> quantile normalization -> Log2 transformed -> Probes aggregated by mean.	Association of RNA Biosignatures With Bacterial Infections in Febrile Infants Aged 60 Days or Younger. JAMA 2016	Prospective observational	Infants aged 60 days or younger evaluated for fever at emergency department.	Blood - Tempus tube
GSE25504	GPL6947	Illumina HumanHT-12 V3.0 expression beadchip	Sepsis	Positive blood culture	Full clinical assessment for early and late symptoms and signs of sepsis followed presentation criteria for neonatal sepsis (included respiratory, cardiovascular, and/or metabolic symptoms, temperature instability, feeding intolerance, lethargy/low tone, jaundice, and/or ill appearance/poor color), and the blood culture was used as the gold standard for diagnosis of sepsis.	A41.89	125	Sepsis bacteremia	25	Healthy Control	35	Robust spline normalised data obtained from GEO.	Whole blood gene expression profiling of neonates with confirmed bacterial sepsis. Genom Data 2015	Prospective case–control	Infants aged 111 days or younger investigated for suspected infection at neonatal unit.	Blood - PAXgene tube
	GPL15158	Codelink 55K Human Array						Sepsis bacteremia	23	Healthy Control	27	RMA-normalized, Log2 transformed data obtained from GEO. Probes aggregated by mean.	Whole blood gene expression profiling of neonates with confirmed bacterial sepsis. Genom Data 2015			
	GPL13667	[HG-U219] Affymetrix Human Genome U219 Array						Sepsis bacteremia	9	Healthy Control	6	RMA-normalized, Log2 transformed data obtained from GEO. Probes aggregated by mean.	Whole blood gene expression profiling of neonates with confirmed bacterial sepsis. Genom Data 2015			
GSE69686	GPL20292	[hGlue_3_0] Custom Affymetrix Human Transcriptome Array	Sepsis and Clinical Sepsis	Mixed (clinical and laboratory)	The decision to evaluate a neonate for sepsis was at the discretion of the attending clinician. See *Supplemental Information*.	A41.89 and A41.9	122	Sepsis bacteremia	64	Healthy Control	58	RMA-normalized, Log2 transformed data obtained from GEO. Probes aggregated by mean.	Postnatal Age Is a Critical Determinant of the Neonatal Host Response to Sepsis. Mol Med 2015	Prospective observational	Infants aged 140 days or younger investigated for suspected infection at neonatal unit.	Blood - PAXgene tube
GSE26378	GPL570	[HG-U133_Plus_2] Affymetrix Human Genome U133 Plus 2.0 Array	Septic shock	IPSCC	International pediatric sepsis consensus conference 2005	R65.21	15	Septic Shock	13	Healthy Control	2	RMA-normalized, Log2 transformed data obtained from GEO. Probes aggregated by mean.	The influence of developmental age on the early transcriptomic response of children with septic shock. Mol Med 2011	Cross-sectional	Children aged 10 years or younger admitted to the pediatric intensive care unit and meeting pediatric-specific criteria for septic shock.	Blood - PAXgene tube
GSE26440	GPL570	[HG-U133_Plus_2] Affymetrix Human Genome U133 Plus 2.0 Array	Septic shock	IPSCC	International pediatric sepsis consensus conference 2005	R65.21	27	Septic Shock	17	Healthy Control	10	RMA-normalized, Log2 transformed data obtained from GEO. Probes aggregated by mean.	Identification of pediatric septic shock subclasses based on genome-wide expression profiling. BMC Med 2009	Cross-sectional	Children aged 10 years or younger admitted to the pediatric intensive care unit and meeting pediatric-specific criteria for septic shock.	Blood - PAXgene tube

IPSCC, International Pediatric Sepsis Consensus Conference.

Description of the title-journal-year of publication of the associated articles, the patient group(s), the total number of patients, the type of study, clinical setting of the study, and sample collection/preservation method for each dataset.

High heterogeneity was evident despite prior individual dataset normalization. Thus, COmbat CO-Normalization Using conTrols (COCONUT) (20), an empirical Bayes normalization-based algorithm, was utilized to co-normalize the merged dataset. In brief, platform-specific control samples were co-normalized to allow for a direct comparison against all case samples. Expression of housekeeping genes (ATP6V1B1 and GAPDH) and those known to be modulated by disease status (CEACAM1 and DYSF) were used to evaluate the normalization performance as previously described (20, 23). Pearson’s correlation was used to assess the prior and post normalized distributions and whether the relationship between the control and case groups was maintained. All subsequent analyses were performed on the log_2_ co-normalized (COCONUT-normalized) merged dataset, unless stated otherwise.

### Patient classification with published gene predictors

FAIM-to-PLAC8 ratio (RG), SeptiCyte Lab (SLS), and Sepsis Metascore (SMS), were calculated as published (11, 14, 15). For others, the mean of the gene markers’ expressions was used. For categorization, each individual is binarized (0 = Control; 1 = Case), using the 3rd quartile of the healthy controls as the threshold for Case. Area under the ROC curves (AUROCs) for each of the gene predictor sets were plotted and confusion matrix and statistics (Caret v.3.45) were used to determine the accuracy, sensitivity, and specificity for the published gene markers.

### Pseudotime analysis

We explored the relationship between all groups on an assumed continuum of disease progression in pseudotime, using the overall gene expression profiles of individual subjects, in Monocle3 (24). In brief, the merged dataset was anti-log_2_ transformed and processed with the function *preprocess_cd()* with num_dim = 50 and reduced via method = UMAP. The earliest principal node was identified with “Healthy Controls” set as the closest vertex index. Genes that were most specifically expressed in each group along the pseudotime trajectory were calculated using *topmarker*() with the following parameters: reference_cells = 1000, marker_test_q_value< 0.01 and specificity ≥ 0.50.

### Differential gene expression analysis and gene ontology enrichment

Differentially expressed genes (DEGs) were determined in a pairwise manner between disease groups or pseudotime clusters using a two-sided Wilcoxon rank-sum test. Significance was denoted by the following thresholds: log_2_FC >1 and Benjamini-Hochberg (BH) adjusted p-value (p.adjust) < 0.05. Gene Ontology (GO) enrichment analysis using *clusterProfile* (25) was performed on the DEGs obtained. In brief, a single gene set per comparison was queried for org.Hs.eg.db GO terms associated with the perturbed biological processes with the following thresholds: minimal gene set size at 3, FDR < 0.05, and dispensability threshold = 0.4 for term redundancy reduction via *simplify()*.

### Immune cell type deconvolution

The relative distribution of 22 different human immune cell types was deconvoluted using LM22 as the reference signature with CIBERSORTx (26) (https://cibersortx.stanford.edu/). A correlation p-value < 0.05 was applied resulting in 288 samples; all but one Septic Shock sample were dropped, thus the group was excluded. Absolute deconvolution of 29 immune cell types was also obtained using the Shiny app (https://github.com/giannimonaco/ABIS) (27). This method has no constraints, hence in case where values were close to zero - due to presumed technical or biological variability - they were set to zero. In case where values were very low negative values due to strong biological or technical variability for the cell type - the cell type was excluded from the analysis.

### Classification performance of gene modules and signatures

Genes deemed important in differentiating disease groups and clusters were combined into modules. Sets of modules were tested for their classification ability. Random forest classification algorithm was performed using MetaboAnalyst 5.0 (28) and significant features (i.e., genes) of importance (mean decrease accuracy > 0.5%) of the classification model were extracted. We computed scores based on the sum of linear expression of the genes selected. For categorization, each individual was binarized (0 = Control; 1 = Case) using the 3rd quartile of the healthy controls as the threshold. The formulas for the scores were 1) log ( $\sum^{\;}\left(pos.reg^{\frac{1}{1.5}}\right)/\sum^{\;}(neg.reg^{0.5}$ )), for the combination of gene modules, and 2) log (ABS( $\sum^{\;}\left(pos.reg)/25^{0.5}\right)\;-\;\sum^{\;}(neg.reg/21^{0.5}$ ))), for the Garnett marker signature. The sensitivity, specificity, and accuracy of each score to classify individuals as Healthy Control or Case (individuals with Bacteremia and Septic Shock) was determined using the ‘caret v.3.45’ package in R.

### Statistical analysis

Unless otherwise stated, Kruskal-Wallis one-way analysis of variance by ranks test was used to determine the difference among multiple groups and Wilcoxon rank-sum test was used for two-group comparisons. Significances are denoted at p.adjust < 0.05.

## Results

### Characteristics of the selected datasets and cohorts

A total of five datasets, encompassing six different microarray platforms, satisfied our selection criteria and were retrieved from public databases (**Table 1**). The datasets were generated from prospective observational (n = 2), prospective case-control (n =1), or cross-sectional studies (n = 2). In all the studies, whole blood was collected and stabilized in RNA-preserving solution (Tempus or PAXgene). We categorized the combined subjects (n = 335) into three groups: Bacteremia (n = 151), Septic Shock (n = 30), and Healthy Controls (n = 154). Age is reported for all datasets, with an overall mean at 18 days (95% CI 15-22). Sex was available for three out of the five datasets and the estimated distribution is 59% male and 41% female. A list of the most common clinical variables is available in **Supplementary File 1** and includes additional information.

### COCONUT normalization circumvented potential biases from microarray platforms, age, and sex while preserving gene expression contrast between healthy controls and patients

High heterogeneity across microarray platforms is a common feature. In the merged dataset, obvious platform and study-specific biases were observed (**Supplementary Figure 1**). Thus, additional normalization was performed using COCONUT (20). Pearson’s correlation (cor = 0.982, p-value < 2.2e-16) and Empirical Cumulative Distribution Function were used to assess the prior and post normalized distributions. The marked difference in overall gene expression profile between the control and case groups (Bacteremia and Septic Shock) was shown to be maintained after this additional normalization (**Supplementary Figure 2**).

**Figure 1:**
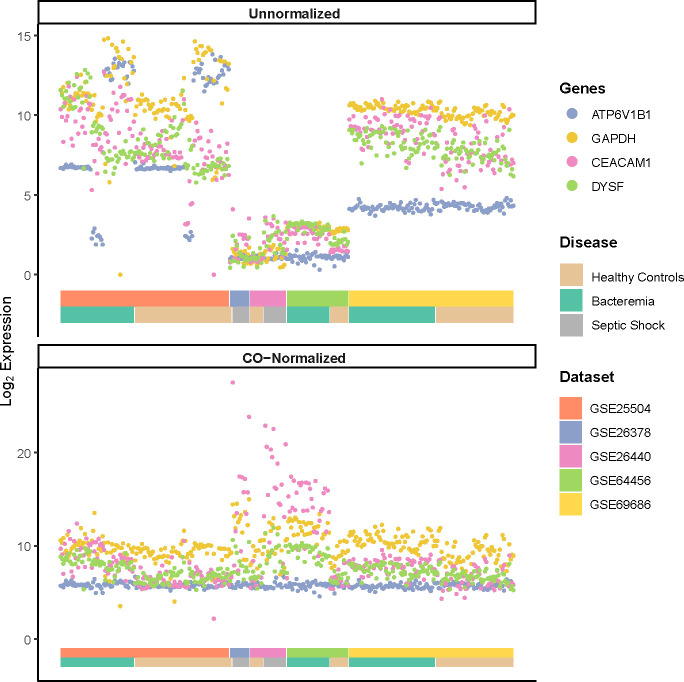
Log_2_ expression of housekeeping genes (ATP6V1B1 and GAPDH) and markers of infections (CEACAM1 and DYSF) pre- and post-normalization using COCONUT. A much smaller variance for the housekeeping genes, after normalization (bottom panel) is seen compared with that of pre-normalization (top panel). The normalization step did not diminish the pronounced increased expression of genes related to infection. The double-layered color-coded bars indicate the source dataset (top bar) and the disease group (bottom bar) and is the same in both top and bottom panels. The legend on the right provides color-coded annotation to the dataset and disease group.

To illustrate the performance of the COCONUT normalization, we visualized the log_2_ expression values of commonly used house-keeping genes (ATP6V1B1, and GAPDH) and genes known to be modulated by infection (CEACAM1 and DYSF) (**Figure 1**) as previously shown (20, 23, 29). A much smaller variance for the housekeeping genes ATP6V1B1 and GAPDH, after normalization (**Figure 1**, bottom panel) was seen compared with that of pre-normalization (**Figure 1**, top panel). The pronounced increased expression of genes related to infection (CEACAM1 and DYSF) were not diminished. Furthermore, the post-normalized data showed a marked reduction of platform bias and better distribution of gene expression among the Healthy Controls. GAPDH expression was additionally assessed, as it has been reported to vary in elderly individuals with sepsis (30), yet frequently used to normalize gene expression in previous studies of neonates with sepsis (12, 31). Principal component analysis (PCA) on the post-normalized data (**Supplementary Figures 1C, D**) also showed marked improvement of gene expression distribution across platforms and Healthy Controls compared with pre-normalization (**Supplementary Figures 1A, B**). In addition, we did not observe notable cluster separations due to the age or sex of the subjects (**Supplementary Figures 1E, F**). Therefore, COCONUT normalization had effectively removed study-specific biases, including the technology platform used, and maximized gene expression profile homogeneity ahead of downstream analyses.

### Published biomarker gene signatures have limited usability for detection of sepsis in infants unless a signature is specifically validated for that population

**Figure 2:**
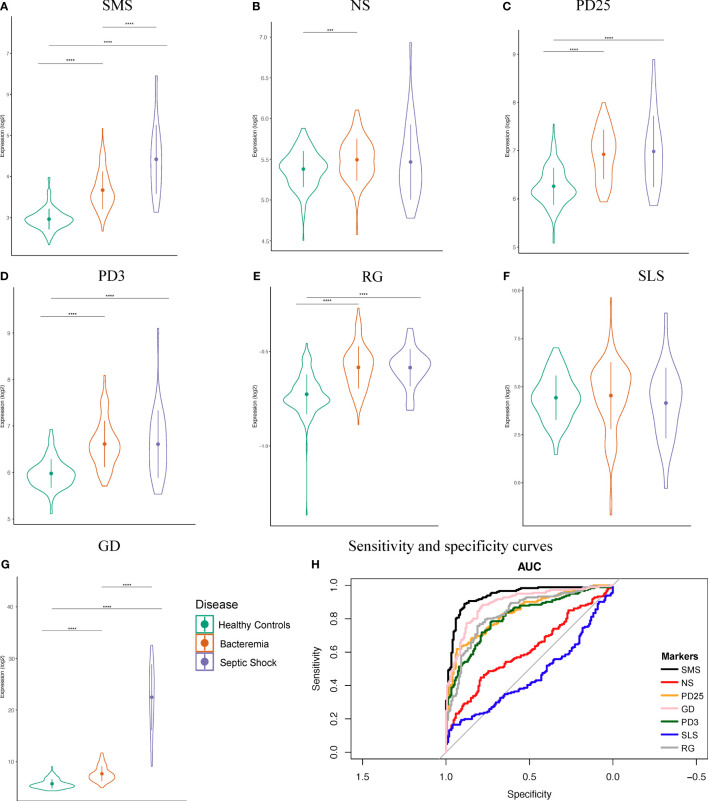
Performance of seven public sepsis marker signatures. **(A–G)** Sepsis Metascore (SMS), 7-gene signature from neonates (NS), 25-gene signature from pediatrics (PD25), 3-gene signature from pediatrics (PD3), FAIM-to-PLAC8 ratio (RG) from adults, SeptiCyte Lab (SLS) from adults, and 6-gene signature from geriatrics (GD). We calculated the individual scores and plotted their distribution, mean, and standard deviation. Patients were grouped as indicated. Kruskal-Wallis one-way analysis of variance by ranks test was used to determine significance between the group means, followed by Wilcoxon Rank test for the specific two-group comparisons at a Bonferroni adjusted p-value < 0.05. Significance is denoted by asterisks: p-value < 0.001 (***), and 0.0001 (****). **(H)** Sensitivity and specificity curves for the performance of seven known sepsis marker signature on our combined dataset comprising of infants.

The first six month of life harbors distinct immune responses that progressively develop into ‘adult’ like, including defense mechanisms against viral and bacterial infections (2, 32). This has meaningful implication for sepsis biomarker signatures developed in cohorts of various age categories. To examine this aspect in our newly merged infant dataset, we assessed the classification performance of seven sets of published sepsis gene signatures determined from various cohorts (**Supplementary Table 1**), namely: Sepsis Metascore (SMS) (11), 7-gene signature from neonates (NS) (12), 25-gene signature from pediatrics (PD25) (13), 3-gene signature from pediatrics (PD3) (21), SeptiCyte Lab (SLS) (14), FAIM-to-PLAC8 ratio (RG) (15), and 6-gene signature from geriatrics (GD) (16). We calculated the signature scores for each of our groups and found significant informative differences about the generalizability of the markers and their usage in wider age groups, such as infants aged 0 to 6 months (**Figures 2 A–G**). The accuracy of SMS is evident by the significant increases in score value for each Case group compared against Healthy Controls, and between Case groups, indicating a good overall capture of disease severity (**Figure 2A**). A similar observation can be made only for the GD signature. On the other hand, the NS signature showed significant difference only between the Healthy Controls and Bacteremia groups. No significant differences were observed with SLS; note that this score was derived from adult cohorts. We then examined the signature performance between all cases combined (either Bacteremia or Septic Shock) vs Healthy Controls. We found that SMS performed the best (accuracy = 85%), followed by GD, RG, PD25, and PD3 (82%, 77%, 75%, 74%, respectively; **Figure 2H**). The performance of NS and SLS were notably less accurate (60% and 56%, respectively). The utility of gene biomarker signature is mostly dependent on the age group from which it was derived and/or validated, highlighting the need to further knowledge of gene expression dynamics in specific cohort such as in infants ≤ 6-months of age.

### A data-driven approach comprehensively delineated the gene expression profile of each patient, revealing distinctive molecular clusters that offer valuable insights into the disease state, particularly for patients with bacteremia

**Figure 3:**
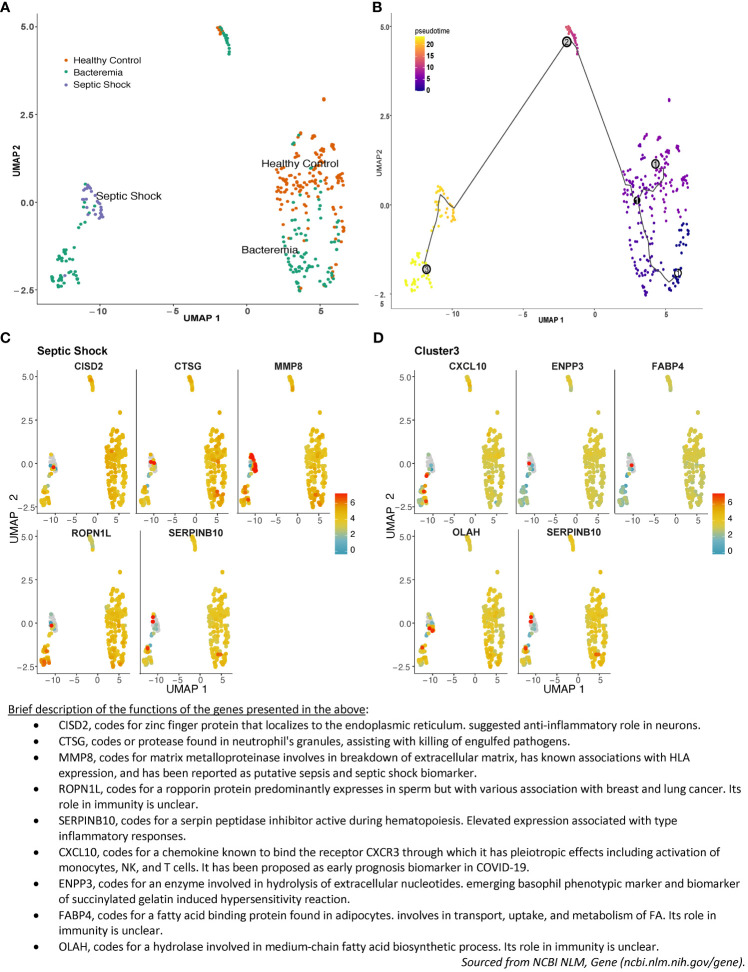
Pseudotime pattern exploration of whole blood transcriptome. Trajectory inference, accomplished through pseudotime analysis using Monocle3 in R, facilitated an unbiased ordering of samples along a trajectory, leveraging similarities in their transcriptome-wide gene expression patterns. This method enabled a comparison between sample groupings generated via pseudotime clustering and those established using standardized clinical disease criteria. In the plotted representation, each dot symbolizes an individual, with its position on the UMAP plot determined by coordinates. The color scheme is either: **(A)** indicative of disease groups (orange for Healthy Controls, turquoise for Bacteremia, and purple for Septic Shock), or **(B)** reflective of disease progression, showcasing pseudotime values and cluster numbers. Three nodes formed Cluster 1 in the lower right quadrant, Cluster 2 is in the center-top, and Cluster 3 is in the lower left quadrant. As such, individuals from the Bacteremia group are spread over the trajectory, while Healthy Controls are mostly segregated to Cluster 1 and Septic Shock are exclusively in Cluster 3. Examples of the expression of the top 5 single gene that best represented this grouping (aka Garnett marker) for **(C)** the Septic Shock and **(D)** Cluster 3. Each gene marker was mapped to the pseudotime plot where each dot represents an individual and the color scale represents gene expression level (log_2_). Thus, each plot allows to visualize how one gene is expressed across the clinical groups. As well, one can assess the range of expression within each pseudotime cluster. For example, SERPINB10 is shown as a marker of Septic Shock and Cluster 3 with predominantly low level of expression. A brief description of the functions of each of the marker shown is listed below the plots. The lists of all markers for all groups and clusters are available in **Supplemantary File 3**.

Sepsis pathogenesis evolves along a continuum of events from the presence of a pathogen in the blood, to counteracting immunosuppression, before deterioration into multiple organ failure, with major changes in cellular processes accompanying pathogenesis. At the molecular level, those changes are preceded by changes in gene expression. Drawing upon the large sample size of the merged studies, we explored the relationship in the overall gene expression of the individual subjects on an assumed continuum of disease progression in pseudotime between all groups. The results showed a pseudotime trajectory with three distinct clusters: Cluster 1 is composed of individuals from the Healthy Controls and Bacteremia groups, Cluster 2 is composed majorly of individuals from the Bacteremia group along with a few from Healthy Controls, and Cluster 3 includes all individuals from the Septic Shock group and some from Bacteremia (**Figures 3A, B**; **Supplementary File 2** for a 3D interactive model). The mixture of individuals with bacteremia and septic shock seen in Cluster 3 suggests a similarity between those individuals at the molecular level and may be indicative of a greater risk of progression towards shock for those clinically categorized as Bacteremia in that cluster. Mapping the expression of gene markers defining a cluster (referred to as Garnett markers) facilitated visualization of the pattern, exemplified for Septic Shock in **Figure 3C** and Cluster 3 in **Figure 3D** (refer to **Supplementary File 3** for lists of all markers for each group and cluster). For instance, a low expression level of SERPINB10 emerged as a marker of Septic Shock and Cluster 3 (**Figures 3C, D**). Notably, three patients exhibited markedly higher SERPINB10 expression than others. MMP8 and CISD2 also distinguished patients with Septic Shock from others within Cluster 3 (**Figure 3D**). Specifically, MMP8 expression was highest in patients with Septic Shock, yet several within the same group displayed the lowest expression levels. These findings advocate for the application of gene signatures or modules where phenotype classification or prediction is not solely based on individual gene expression but on a collective of genes, whether they function in concert or independently. In subsequent analyses, as detailed below, we evaluated the performance of the Garnett markers in classifying individuals into clinical groups.

### Differential gene expression analysis highlighted more pronounced perturbations in septic shock, primarily centered around neutrophil activation, while changes in bacteremia primarily revolved around T cell activation

In order to identify those gene markers that are the most representative of disease progression, we performed differential gene expression analysis based on groups formed either by clinical disease status or by pseudotime clustering (**Supplemntary Figure 3**). This comparative analytical approach allowed for an expansion on the results generated through pseudotime clustering, and to contrast the DEG’s identified with those generated based on standardized clinical disease criteria.

**Figure 4:**
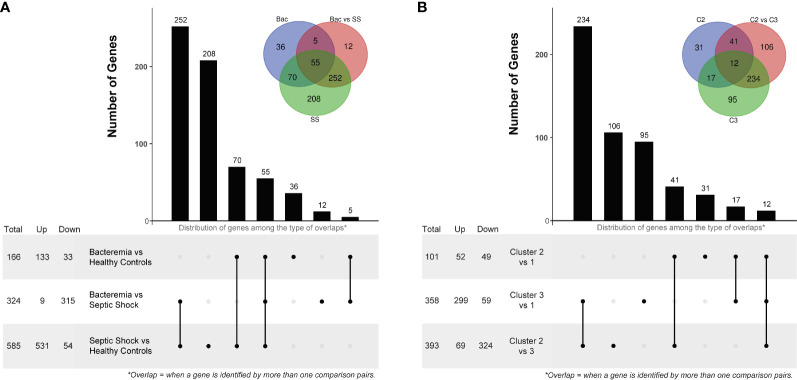
Differentially Expressed Gene (DEG) analysis comparing disease groups and pseudotime clusters. The histogram and associated bottom panel depict the number of DEGs that were commonly identified (i.e., overlap) by the different disease group **(A)** and cluster **(B)** comparisons. With each histogram plot, a traditional Venn diagram shows the same information, where the number of DEGs is indicated in the intersecting circles. For example, in panel A, there are 36 DEGs that were only identified by comparing the Bacteremia group against Healthy Controls, this is indicated above the bar in the histogram and in the non-intersecting area of the inset Venn diagram (blue circle labelled “Bac”). DEG analysis was performed using two-sided Wilcox Rank test with the following significance thresholds: |log_2_FC|> 1 and p.adjust < 0.05. For the inset Venn diagram, the abbreviations are: Bac, Bacteremia vs Healthy Controls; Bac vs SS, Bacteremia vs Septic Shock; SS, Septic Shock vs Healthy Controls; C2, Cluster 2 vs 1; C2 vs C3, Cluster 2 vs 3; and C3, Cluster 3 vs 1.

The first differential gene expression analysis was performed on all pair-wise comparisons of the two case groups and Healthy Controls (**Supplementary Figures 4A–C** for volcano plots and **Supplementary File 4** for the full lists). The number of DEGs and overlaps among disease groups is summarized in **Figure 4A**. Of note, among the top 10 common DEGs, modulation within the Septic Shock group showed the greatest log_2_FC values, especially for up-regulated DEGs (**Supplementary Table 2**). When looking at the DEGs uniquely modulated (**Supplementary Table 3**), those in the Bacteremia group involved functions related to “immune response to external stimuli”, “leukocyte migration”, and T cell differentiation/activation. Strikingly, the down regulations of CD3E, CD3G, ZAP70, LCK and CD5 represent a gene module specifically associated with T cell activation. DEGs uniquely modulated in the Septic Shock group are largely involved in functions related to “leukocytes/granulocytes activation”, in particular neutrophils, and “regulation of exocytosis”. When comparing Bacteremia gene expression against Septic Shock (as opposed to comparing against Healthy Controls), 12 DEGs were unique among the 324 identified and the majority were related to nuclear/chromatin processes; 11 were down- and 1 was up-regulated in Bacteremia compared with Septic Shock. Notably, the single upregulated gene was CXCL10 (linear FC of 2.4), a multifunctional chemokine that is potently induced in response to IFN-gamma signaling (33).

The second differential gene expression analysis was performed using the clusters formed in the pseudotime analysis with Cluster 1 as the reference (**Supplementary Figures 4D–F** for volcano plots and **Supplementary File 4** for the full lists). The number of DEGs and overlap among clusters is summarized in **Figure 4B**. Of note, among the top 10 common DEGs, there were several instances where a reversal in the direction of changes between clusters was observed (**Supplementary Table 2**); for example, CKAP4 and ROPN1L were down-regulated in Cluster 2 but strongly up-regulated in Cluster 3. When looking at the DEGs uniquely modulated (**Supplementary Table 3**), those in the Cluster 2 involved functions related to “protein localization to the endoplasmic reticulum” and “mRNA catabolism”. In Cluster 3, the DEGs involved functions related to “leukocytes/granulocytes activation”, in particular neutrophils, and “regulation of exocytosis” as similarly observed for the Septic Shock group. When comparing Cluster 2 gene expression against Cluster 3, 106 DEGs were unique among the 393 identified; 81 were down- and 25 were up-regulated. The functional roles of these genes were mainly related to “lipid metabolism”, “carboxylic acid metabolism”, and “activin receptor signaling pathway”. Interestingly, CXCL10 was significantly upregulated (linear FC of 2.1) only when comparing Cluster 3 against Cluster 1, possibly corresponding to the contribution of individuals with bacteremia whose transcriptomic profiles indicated a transition towards septic shock.

The comparison of the data-driven clusters provided more granular information about the molecular changes that accompany the states of bacteremia vs sepsis. Indeed, comparing Cluster 2, mostly patients with bacteremia, to Cluster 3, a mixture of patients with bacteremia and septic shock, we found 106 DEGs unique to this comparison; in contrast, there were only 12 unique DEGs between the Bacteremia and Septic Shock groups. Thus, this list of DEGs is particularly interesting to investigate biomarker genes associated with the process of advancing disease severity.

### Gene ontology enrichment analysis revealed significant enrichment of coagulation with disease progression to septic shock

To gain further understanding of the biological processes perturbed during sepsis, we performed GO enrichment analysis separately on the up- and down- regulated DEGs from each list generated either from comparing the disease groups or the pseudotime clusters (**Supplementary Figure 3**). The full lists of enriched GO terms for all the comparisons are provided in **Supplementary File 5**.

**Figure 5:**
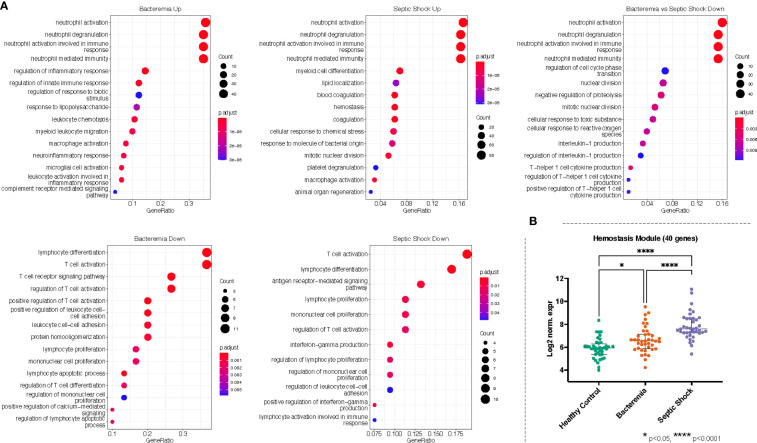
Gene ontology (GO) enrichment analysis derived from the up- and down-regulated DEGs lists from disease groups as indicated. **(A)** The plots show the enriched GO terms, with the adjusted p-value as the color and the number genes that took part in the enrichment of the terms as the size of the circles. All plots show the top GO 15 terms by p.adjust rank. **(B)** Genes belonging to hemostasis related processes (GO:0007596, GO:0007599, GO:0050817, GO:0002576) were combined, forming a module of 40 genes, and the log_2_ normalized expression values of all 40 genes were plotted for each group. Kruskal-Wallis with Dunn’s multiple comparisons test was used to assess significant difference between group as indicated.

Against the background gene set (total number of genes included in the merged dataset, 8466 genes), 121 and 16 GO terms were enriched in Bacteremia using the up and down lists of DEGs, respectively. In comparison, there were 175 and 12 GO terms enriched in Septic Shock using the up and down lists of DEGs, respectively. A succinct summary of the key enrichment categories is shown in **Table 2** and the top result for each comparison is shown in **Figure 5A**. Interestingly, we found that GO terms related to coagulation were markedly enriched in the Septic Shock group (see ‘Septic Shock Up’ plot in **Figure 5A**) and upon inspecting the log_2_ normalized expression of all the genes that contributed to the enrichment of hemostasis-related processes, we found a distinct hemostatic signature (**Figure 5B**). We then investigated GO enrichment using the list of DEGs that came from comparing Bacteremia against Septic Shock. There was no GO enrichment for the up-DEGs list (only 9 DEGs were identified). From the down-DEGs list, we observed major enrichment of “neutrophils activation”, indicating that the state of septic shock involves a significant increase in neutrophils activity.

**Table 2:** Summary of GO term enrichment that predominates in each group/cluster using up- and down-regulated gene sets as indicated.

Representative enriched GO terms - comparing among disease groups	
Derived from up-regulated DEGs in Bacteremia (vs. Healthy Controls)	Derived from up-regulated DEGs in Septic Shock (vs. Healthy Controls)
● Inflammatory/innate immune response (GO:0050727, GO:0045088, GO:0002269)	● Hemostasis processes (GO:0007596, GO:0007599, GO:0050817, GO:0002576)
● Myeloid leukocyte migration (GO:0030595, GO:0097529)	● Myeloid cell differentiation (GO:0030099)
● Neuroinflammatory (microglia) response (GO:0001774, GO:0150076)	● Response to stress (GO:0062197)
	● Mitotic division (GO:0140014)
Derived from down-regulated DEGs in Bacteremia (vs. Healthy Controls)	Derived from down-regulated DEGs in Septic Shock (vs. Healthy Controls)
● Lymphocyte apoptotic process (GO:0070227, GO:0070228)	● IFN-gamma production (GO:0032609, GO:0032729)
● Regulation of calcium-mediated signaling (GO:0050850)	
Representative enriched GO terms - comparing among clusters	
Derived from up-regulated DEGs in cluster 2 (vs. cluster 1)	Derived from up-regulated DEGs in cluster 3 (vs. cluster 1)
● Erythrocyte homeostasis (GO:0006779, GO:0033014, GO:0055072, GO:0048821, GO:0006778, GO:0034101)	● Neutrophil activation (GO:0042119, GO:0043312, GO:0002283, GO:0002446)
● Cellular oxidant detoxication (GO:0098869, GO:0098754)	● Inflammatory response to external stimuli (GO:0002237, GO:0032102, GO:0071216, GO:0031667, GO:0002831)
Derived from down-regulated DEGs in cluster 2 (vs. cluster 1)	Derived from down-regulated DEGs in cluster 3 (vs. cluster 1)
● Translation-associated targeting to membrane Protein targeting to ER (GO:0006614, GO:0006613, GO:0006413 - GO:0045047, GO:0072599)	● T cell activation/proliferation (GO:0042110 GO:0030098, GO:0050852, GO:0050863, GO:0050870, GO:0042129, GO:0046651, GO:0002285)
	● Leukocyte cell-cell adhesion (GO:1903037, GO:0007159)
	● IFN-gamma production (GO:0032609, GO:0032729)
	● Translation with protein targeting to membrane (GO:0006614, GO:0006413)

The name of each group of GO was given to best represent the categories encompassed, with exact GO ID provided in parentheses. Circles represent the average log_2_FC (scaled 1/10) of the genes that contribute to the enrichment; red and blue indicate enrichment from lists of up- and down-regulated genes, respectively. All GO terms considered for this summary were enriched with p.adjust value< 0.001. Underlined words are to indicated divisions.

**Figure 6:**
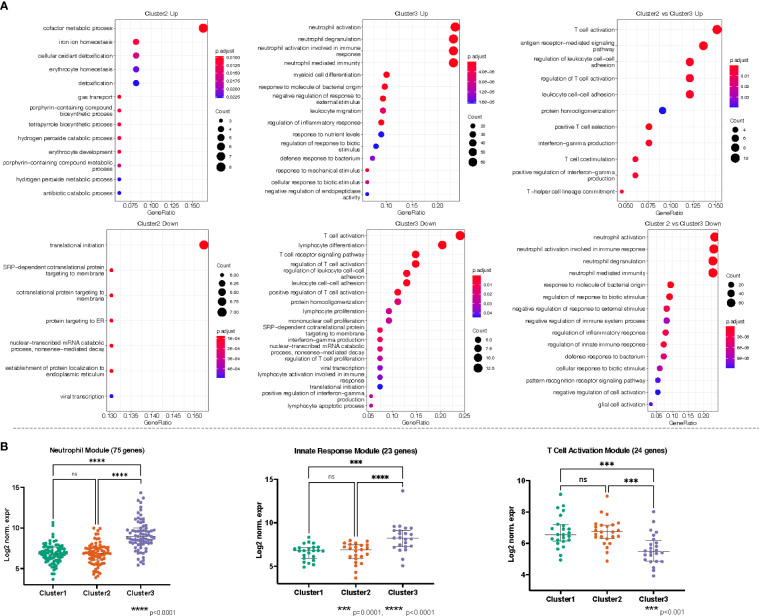
**(A)** Gene ontology (GO) enrichment analysis derived from the up- and down-regulated DEGs lists from pseudotime clusters as indicated. The plots show the enriched GO terms, with the adjusted p-value as the color and the number genes that took part in the enrichment of the terms as the size of the dot. All plots show the top GO 15 terms by p.adjust rank, except “Cluster 3 Down” which shows all 19 GO terms enriched for that gene set, and “Cluster 2 vs Cluster 3 Up” which shows 11 GO terms enriched for that gene set. **(B)** Genes belonging to neutrophils (GO:0042119, GO:0043312, GO:0002283, GO:0002446), innate immune responses (GO:0050727, GO:0045088, GO:0002269), and T cell activation/receptor (GO:0042110, GO:0030098, GO:0050852, GO:0050863, GO:0050870, GO:0042129, GO:0046651, GO:0002285) related processes were independently combined to form modules of 75, 23, and 24 genes, respectively. The log_2_ normalized expression values of the genes in each module were plotted for each cluster. Kruskal-Wallis with Dunn’s multiple comparisons test was used to assess significant difference between group as indicated.

Recalling the data-driven approach, three clusters were generated: Cluster 1 is mainly composed of Healthy Controls, Cluster 2 is composed majorly of individuals in the Bacteremia group, and Cluster 3 is a mix of individuals with Bacteremia and Septic Shock. With Cluster 1 as reference, the DEGs obtained with cluster 2 enriched 13 (using up DEGs) and 7 (using down DEGs) GO terms. For cluster 3, there were 171 (using up DEGs) and 19 (using down DEGs) enriched GO terms (see **Table 2** for brief summary and **Figure 6A** for top results of each comparison). When comparing Cluster 2 against Cluster 3, the up-DEGs list enriched GO terms predominantly pertaining to “T cell activation”, “receptor-mediated signaling”, and “IFN-gamma production”. The down-DEGs list markedly enriched terms pertaining to “neutrophil activation”, “responses to external/bacterial stimuli”, “innate immune responses” (**Figure 6A**). Upon investigating the log_2_ normalized expression of all the genes that contributed to the enrichment of neutrophils, innate immune responses, and T cell activation, we found that Cluster 3 was clearly distinguishable from the other clusters (**Figure 6B**), as well, the Septic Shock group was also significantly different compared with Bacteremia and Healthy Controls (**Supplementary Figure 5**).

The enrichment of biological processes related to neutrophil activation in Septic Shock and T cell activation in Bacteremia recapitulates the findings from the DEG analysis. Further, we found that coagulation is progressively enriched as disease severity increases.

### Performance assessment of gene modules and Garnett markers showed accuracy above 80% to classify subjects in their respective disease group

To follow up with the gene modules identified after our exhaustive GO enrichment analyses, we tested their ability to classify patients by disease group. First, we used a random forest algorithm to extract the significant features (i.e., genes) of importance that contribute the most to the classification model. The combination of hemostasis, neutrophils, innate immune responses, and T cell activation modules, a total of 142 genes, returned 28 significant genes of importance with mean decrease accuracy > 0.5%. A gene expression score was computed with those 28 genes and ROC curve analysis indicated a classification accuracy of 0.813 (0.747 sensitivity, 0.878 specificity) (**Supplementary Figure 6A**). We also used the Garnett markers identified with pseudotime analysis as the input for the random forest analysis. Using the score calculated from the 46 significant Garnet marker, we obtained an accuracy of 0.818 (sensitivity = 0.747, specificity = 0.879) (**Supplementary Figure 6B**). These encouraging preliminary findings add support to the tactics we used to generate infants-appropriate gene targets and hold promise to improve and refine sepsis biomarkers.

### Immune cell deconvolution of the bulk RNA expression data emphasized the reduced involvement of B cell and multiple T cell subtypes

**Figure 7:**
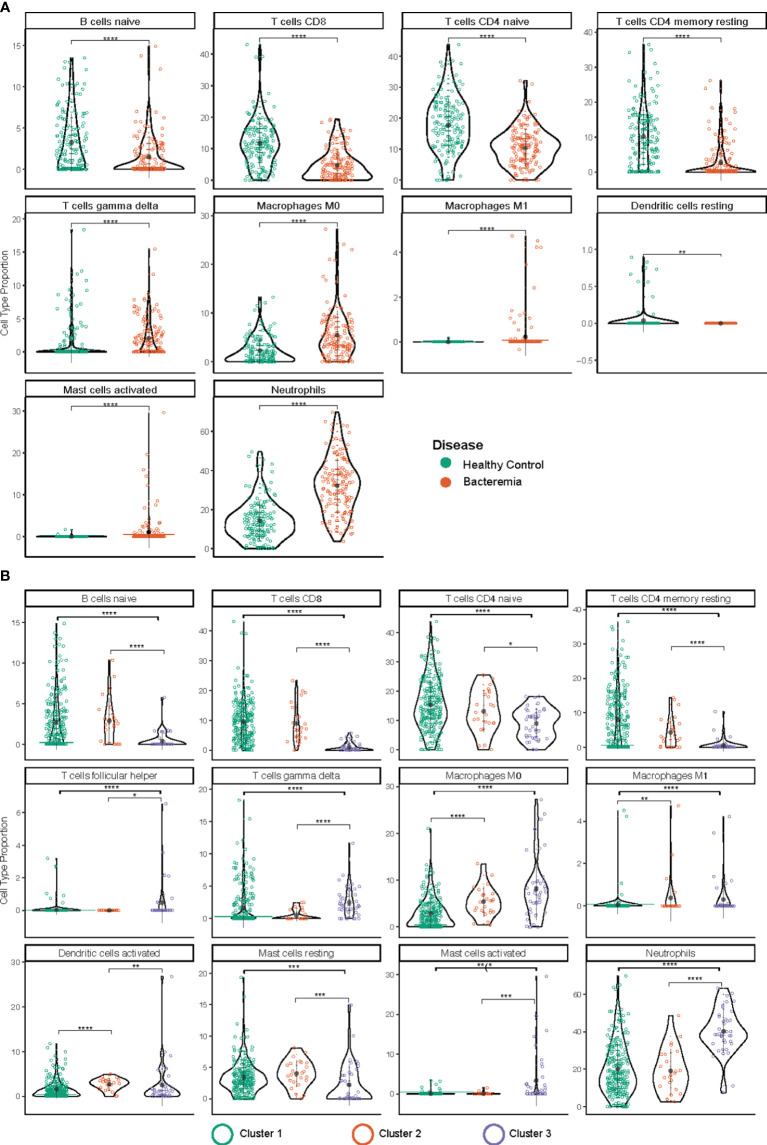
Immune cell type deconvolution by CIBERSORT. The cell type is indicated at the top of each graph. The y-axis denotes the relative proportion of cell type (%). The color of the dots corresponds to the legend color for the different disease groups **(A)** and pseudotime clusters **(B)**. Depicted are mean with standard deviation and statistical significance by Kruskal Wallis t test is indicated by asterisk; p.adjust < 0.05 (*), 0.01 (**), 0.001 (***), and 0.0001 (****). The markers used in the reference dataset to define each cell type are: CD19+CD27- IgG/A- for B cells naïve; CD3, CD8, CD45RA for T cells CD8; CD4+ for T cells CD4 naïve; CD45RO^high^ for T cells CD4 memory resting; T cells gamma delta (not reported); macrophage M0 (none known/reported; identified by morphology and phagocytic capability); macrophage M1 (none known/reported; identified by morphology and phagocytic capability); mast cells resting (n/a); for mast cells activated (n/a); and CD62L for neutrophils.

The immune system is a heterogeneous mixture of cells. Disease can affect both cell subsets and proportions of cells present. Recently developed bioinformatic tools allow the extraction of a wealth of information about cell type composition, a process called deconvolution, using bulk transcriptomic data, whether RNA-Seq or microarray-based. Thus, we performed immune cell deconvolution of the bulk RNA expression data utilizing the CIBERSORT analytical pipeline (34). CIBERSORT has shown good validation with flow cytometry for major T and B lymphocytes (35, 36). The results from CIBERSORT are expressed as the relative proportion of cell type per sample, such that the total of all calculated proportions adds up to 100% (an overview of the results is shown in **Supplementary Figure 7A**). After filtering samples with a modelling fit p-value threshold < 0.05, we retained 149 and 139 samples belonging to the Healthy Controls and Bacteremia, respectively, and 223, 24, and 41 belonging to Cluster 1, 2, and 3, respectively. All but one individual from the Septic Shock group were above the threshold, thus the latter group was omitted from the analysis. **Figure 7** shows the relative distribution of the immune cell subsets that were determined to be significantly different between the disease state groups or pseudotime clusters.

Amongst the lymphocyte subsets the relative proportion of B cell (B naïve) and T cell subtypes (CD8, and CD4 naïve, and CD4 memory) were found to be lower in Bacteremia compared with Healthy Controls. Similar trends were observed when comparing the pseudotime clusters with Cluster 3 having the lowest proportions. Interestingly, the proportion of gdT cells was significantly higher in Cluster 3 compared with Cluster 2 and Cluster 1, suggesting a strong “engagement” of these pro-inflammatory effector cells.

Among myeloid cells, neutrophils were significantly increased in Bacteremia compared with Healthy Controls, and Cluster 3 compared with Cluster 2 and Cluster 1. This is concordant with the DEGs and GO enrichment analyses mentioned above.

While the proportion of monocytes showed no differences between any groups (not shown), the proportions of M0 macrophages (undifferentiated) were significantly increased in Bacteremia compared with Healthy Controls and were also significantly higher in Cluster 2 and 3, in comparison to Cluster 1.

For mast cells, clearer insights were gained by comparing the clusters. Results showed that resting mast cells were significantly lower and activated mast cells significantly higher in Cluster 3 compared with Cluster 2 and Cluster 1, suggesting that they contribute to the exacerbation of the inflammatory response. However, mast cells constitute a small proportion of cells present (~0.01%), thus the observed frequency > 5% in a few individuals could represent artefact of the analysis.

To support the results generated with CIBERSORT, absolute deconvolution of 29 immune cell types was also performed using ABIS (https://github.com/giannimonaco/ABIS). We found significant differences in the proportion of cell types identified (6 out of 11 types assessed) between disease groups and clusters, including B cells, T cells, and neutrophils. CIBERSORT provided greater granularity in terms of cell types assessed (13 out of 22 significantly different proportion), nevertheless, the finding for major cell populations were concordant between both methodologies (**Supplementary Figure 7B**).

Using immune cell deconvolution, we added another dimension to our transcriptomic analyses and complemented the findings from DEGs and GO enrichment regarding the role of neutrophils, naïve B cells, and several subtypes of T cells.

## Discussion

Despite the advent of modern protocols for sepsis recognition and screening, accurate diagnosis and management of sepsis under hospital conditions to prevent progression to septic shock in infants remain a key area of concern (37). As such, there is immense value for determining a blood borne transcriptomic marker signature for sepsis, either for diagnosis, monitoring, or predictive purposes. Ultimately, a biomarker gene signature, composed of 5 to 10 targets and whose expression is tested from whole blood, would have the advantage to be time- and cost-effective. As a first step in that direction, mining of public datasets serve as a valuable resource for achieving increased statistical power and encompassing various clinical settings (11, 20). Furthermore, with sepsis in young infants, a systematic evaluation of multiple studies is highly desirable due to sample scarcity. In this study, we established a coherent compilation of infant-specific transcriptomic datasets. This compilation serves to bolster the identification and development of sepsis biomarkers while concurrently enhancing our comprehension of the molecular and biological alterations occurring during sepsis in infants.

Merging data from multiple studies bears increased data heterogeneity, discrepant variances, and variable demographic characteristics, in addition to the inherent differences of the various sequencing platforms. To minimize the limitations, we 1) curated studies from similar clinical backgrounds and blood RNA preservation methods, 2) employed COCONUT to minimize study biases (20), and 3) focused our analysis on the common genes across datasets, mitigating the putative biases due to probe composition differences across platforms. Moreover, we used the Wilcoxon rank-sum test to determine the differentially expressed genes between cases and controls, as conventional methodology such as DESeq2 (38) would not have been appropriate for post-transformed datasets. Indeed, the DESeq2 model internally corrects for library size, so transformed or normalized values such as counts scaled by library size should not be used as input (38). In addition, compared to other bioinformatics methods, the Wilcoxon rank-sum test is the best suited for DEGs between two conditions using human transcriptomic data (39).

Testing of published sepsis gene signatures with our merged datasets showed a high degree of accuracy for only one of the validated signatures (SMS), and highlighted the narrower utility of other signatures for use in detection of infant sepsis. The heterogeneous performance between the published gene signatures is likely an indicator of age-specific processes, as the first 6 months of life is a period subject to many immunological adaptations (32, 40, 41). However, we cannot completely disregard the differences in clinical settings and cohort characteristics between those studies and ours. As such, the 7-Genes in neonates signature (NS) had a poor degree of DEG overlap and performed poorly in predicting sepsis from controls in our infant dataset. In contrast, SMS and the 6-Genes geriatric gene markers (GD) both exhibited a high degree of overlap with DEGs generated in our analysis and have the highest accuracy and precision in differentiating cases from controls. The concordance with the geriatric markers suggests similarities between the immature immune state of infants and that of an immunosenescent state present in the elderly; an intriguing correlation that potentially warrants further investigation.

Trajectory inference (TI) methods, such as pseudotime analysis, order samples along a trajectory based on similarities in their expression patterns. TI methods offer an unbiased and transcriptome-wide understanding of a dynamic process (42). By ordering each cell according to its progress along a learned trajectory, where the total length is defined in terms of the total amount of transcriptional change, the approach helps understand the sequence of regulatory changes that occur from one state to another. The choice of using Monocle as TI method was driven by the anticipated trajectory topology in the data and usability of the methods. Monocle projects the data into a low-dimensional space and uses UMAP instead of t-SNE which allows preservation of longer-range distance relationships (24, 43). Here, the concordance of increasing pseudotime mapped with disease severity. Interestingly, individuals from the Bacteremia group spanned across the continuum, indicating that the transcriptional profiles are widespread within this clinically-categorized group. Cluster 2 was composed mostly of Bacteremia individuals and is distinctively mapped midway between Healthy Controls and Septic Shock (**Figure 3A**). Since we did not have exhaustive clinical histories for the patients, we cannot rule out associations with specific clinical symptoms and thus represents an aspect of our study that warrants further evaluations. Nevertheless, our findings illustrate that molecular analyses can assist in delineating sepsis disease states in various clinical scenarios. Indeed, as the definition for clinical diagnosis of sepsis varies substantially in the literature, developing a practical molecular tool to help classify sepsis disease states is especially useful and a worthy endeavor.

Our in-depth analyses of gene regulations among the disease groups and pseudotime clustering provided insights into the underlying biological perturbations of sepsis progression in infants. Based on the number of DEGs and the extent of their modulations, we observed a lower level of molecular perturbations in subjects with Bacteremia as compared with the Septic Shock group. Notably, GO enrichment results highlighted the predominance of pro-inflammatory responses and T cell activation during bacteremia. In stark contrast, during septic shock, T cell activation/proliferation and IFN-gamma production were strongly downregulated. Furthermore, our Gene Ontology (GO) enrichment analysis revealed a growing engagement of hemostasis processes as disease states advance to septic shock. The outcomes of immune cell deconvolution not only substantiated the aforementioned findings but also aligned with prior research indicating associations between sepsis, progression to septic shock, and phenomena such as T cell exhaustion (44, 45), B cell dysfunction (46) and heightened rates of apoptosis in both the B and T cell compartments (47).

The central role of neutrophils in sepsis pathogenesis is highlighted by the gene ontology analysis performed on the common DEGs. We found additional enriched processes related to cellular mobilization and stress responses, which are consistent with innate responses to bacterial infections (48, 49). In the Septic Shock group, not only hemostasis and myeloid activation were clearly significantly enriched, but cell cycle processes were also enriched and could be a consequence of increased catabolic activity tied to enhanced cellular stress (50, 51). Taken all the results together, the progression toward septic shock can potentially be tracked by induction of genes involved in the complement system, coagulation, platelet degranulation, neutrophil activation (greater magnitude), and cell cycle transition. Our preliminary assessment of classification performance using the gene modules yielded accuracy > 0.8, however the relatively small sample size in the septic shock group precluded the formation of testing set.

IFN-gamma is a key inflammatory mediator in the establishment of sepsis and has been shown to work in synergy with TNF-alpha to induce a cytokine storm (52). In a lipopolysaccharide-induced sepsis mouse model, Karki et al. (2021) showed that neutralizing both cytokines drastically improved survival (52). This cytokine synergy, and possibly others yet to be discovered, could contribute to disease progression if consistently maintained. This rationale could explain the wide distribution of the transcriptomic profiles within the Bacteremia group, as the concerted activation of IFN-gamma, TNF-alpha, and related pathways would be suppressed among those in the category with transcriptomic profiles most similar to Healthy Controls (Cluster 1), low in those Bacteremia group members that formed Cluster 2, and high in those found in Cluster 3. We speculate that the persistent presence (time dependent factor) and elevated concentrations (quantitative factor) of both cytokines may define a molecular threshold that regulates the progression towards deterioration from bacteremia to septic shock states. We have seen CXCL10 upregulated in Bacteremia and individuals in the Bacteremia group whose transcriptomic profiles indicated a transition towards septic shock. However, the expression of genes related to IFN-gamma production were downregulated in Septic Shock. In the situation where adaptive immunity is declining, NK and NKT are other strong producers of IFN-gamma (53) that could induce monocytes to secrete CXCL10, which can then exert its chemoattractant role on monocytes/macrophages, NK cells, dendritic cells, and T cells (54) (under these conditions T cells may be responsive to chemotaxis, even where their activity is reduced). Altogether, this environment may tilt the immune balance toward a septic shock state. To strengthen our results and enable further research, we constructed comprehensive cytokine profiles, encompassing both cytokines and chemokines, extracted from our list of co-normalized genes. We then proceeded to showcase the outcomes, delineating the expression patterns of the identified 70 cytokine genes across different disease groups and pseudotime clusters. These results are visually depicted in **Supplementary Figure 8**.

## Clinical significance and conclusion

The current unmet need in infant sepsis diagnostics goes beyond distinguishing sepsis from healthy controls, but rather the ability to differentiate patients along the continuum of progression from bacteremia to sepsis and shock with the goal to be used complementarily with clinical examination. The findings from this comprehensive analysis of infant-specific transcriptomic datasets hold significant clinical implications for the diagnosis and management of sepsis in infants. By merging data from diverse studies and employing robust normalization methods, we have established a reliable compilation capturing the molecular landscape across different disease states, from bacteremia to septic shock. Pseudotime analysis reveals distinct molecular clusters aligning with disease progression, shedding light on potential risk transitions for patients clinically categorized as Bacteremia. Our findings highlighted that disease progression is accompanied by increasing pro-inflammatory responses, especially via neutrophil activity, declining T cells activation and relative proportions, and increasing involvement of hemostasis-related processes. Immune cell deconvolution provided a snapshot of immune cell proportions that corroborated with our findings from gene expression analysis. Collectively, these results offer a valuable foundation for advancing our understanding of sepsis in infants, providing insights into molecular alterations, immune responses, and disease progression, laying the groundwork for potential diagnostic and therapeutic advancements in this critical clinical context. A final note, future studies in infants of 6-month of age or younger should be conducted to capture extensive presentation symptoms and clinical data, including innate immune responses (multiplex cytokine panels, neutrophils activation markers) and hemodynamic (coagulation and fibrinolysis factors, complement system) parameters, to enhance the integration of transcriptomic, gene ontology, and pseudotime information.

## Data availability statement

Publicly available datasets were analyzed in this study. This data can be found here: Datasets ID# GSE64456, GSE25504, GSE69686, GSE26378, GSE26440. Datasets were obtained and explored with NCBI Gene Expression Omnibus (GEO) at (https://www.ncbi.nlm.nih.gov/geo/ and SysInflam HuDB at http://sepsis.gxbsidra.org/dm3/geneBrowser/list.

## Ethics statement

Ethical approval was not required for the study involving humans in accordance with the local legislation and institutional requirements. Written informed consent to participate in this study was not required from the participants or the participants’ legal guardians/next of kin in accordance with the national legislation and the institutional requirements.

## Author contributions

SH: Conceptualization, Data curation, Formal analysis, Investigation, Methodology, Validation, Visualization, Writing – original draft, Writing – review & editing. MT: Data curation, Formal analysis, Writing – review & editing. PE: Funding acquisition, Investigation, Resources, Writing – review & editing. NV: Conceptualization, Data curation, Formal analysis, Funding acquisition, Investigation, Methodology, Resources, Visualization, Writing – original draft, Writing – review & editing. MG: Conceptualization, Data curation, Formal analysis, Investigation, Methodology, Project administration, Validation, Visualization, Writing – original draft, Writing – review & editing.

## Supplementary material

Supplementary Figure 1 Multivariate analyses of the merged dataset pre- and post-COCONUT normalization. **(A)** In the pre-normalization data, gene expression profiles are separated by the type of microarray platform and data series. **(B)** Conversely, the pre-normalized data show weak separation based on disease category. **(C)** Post-normalization, we achieved a more uniform distribution of gene expression across series and platforms and **(D)** enhanced the separation between disease groups. The global gene expression does not show effects of the **(E)** age and **(F)** sex of the subjects.

Supplementary Figure 2 The relationship between cases and controls is preserved. Pearson’s correlation (cor = 0.982, p-value< 2.2e-16) and Empirical Cumulative Distribution Function were used to assess the prior and post normalized distributions and the relationships between the control and case groups. The y-axis denotes the data percentile and can be interpreted as the probability of an event at the intersection with the x-axis value. The x-axis represents the observed values.

Supplementary Figure 3 Workflow of the comparative analysis approach taken to delineate markers of disease severity.

Supplementary Figure 4 Differential gene expression analysis comparing disease groups and pseudotime clusters. **(A, B)** Volcano plots representation of DEGs in each group against Healthy Controls and **(C)** for Bacteremia against Septic Shock. D-E) Volcano plots representation of DEGs in each cluster against #1 and F) for Cluster 2 against Cluster 3. Analyses was performed using two-sided Wilcox Rank test. Significance of the DEGs were denoted by the following thresholds: |log_2_FC| > 1 and p.adjust < 0.05.

Supplementary Figure 5 Gene expression pattern of important modules. Genes belonging to neutrophils (GO:0042119, GO:0043312, GO:0002283, GO:0002446), innate immune responses (GO:0050727, GO:0045088, GO:0002269), T cell activation/receptor (GO:0042110, GO:0030098, GO:0050852, GO:0050863, GO:0050870, GO:0042129, GO:0046651, GO:0002285), and hemostasis (GO:0007596, GO:0007599, GO:0050817, GO:0002576) related processes were independently combined to form modules of 75, 23, 24, and 40 genes, respectively. The log_2_ normalized expression values of the genes in each module were plotted for each disease group. Kruskal-Wallis with Dunn’s multiple comparisons test was used to assess significant difference between group as indicated.

Supplementary Figure 6 ROC curve analysis derived from **(A)** The combination of hemostasis, neutrophils, innate immune responses, and T cell activation modules and **(B)** Garnet marker signature.

Supplementary Figure 7 Summary of the immune cell deconvolution. Deconvolution from bulk RNA expression data following the approach by **(A)** CIBERSORT using the LM22 single-cell reference dataset and **(B)** ABsolute Immune Signal (ABIS) deconvolution (available at https://giannimonaco.shinyapps.io/ABIS/). Both results are expressed as the relative proportion of cell type per sample (such that the total of all calculated proportions adds up to 100%). Groups are labelled according to the inset and the asterisk denotes where at least one pair-wise comparison among groups reached significance.

Supplementary Figure 8 Profiles of cytokine, including chemokine, coding genes identified among our co-normalized genes. Each dot represents the log2 normalized expression values. The significance of differences between groups was assessed using the Kruskal-Wallis test with Dunn’s multiple comparisons correction.

Supplementary Table 1 Assessment of selected public sepsis gene signatures. Description of source articles, year published, related dataset ID number, age of the subject evaluated, and gene list that composed each signature.

Supplementary Table 2 Varying degree of modulation for the commonly perturbed genes among the disease groups against Healthy Controls (top panel) and the pseudotime clusters against Cluster 1. Shown is the log_2_FC values of the top 10 up- and down-DEGs which are ranked based on the Bacteremia group (out of 125 common DEGs) or Cluster 2 (out of 29 common DEGs).

Supplementary Table 3 Top 10 unique up- and down-regulated genes, by fold-changes, in the Bacteremia and Septic Shock groups when compared with Healthy Controls (top panel) and in pseudotime clusters 2 and 3 when compared to cluster 1 (bottom panel). Shown in parentheses are log_2_FC values.

Supplementary File 1 List of accessible clinical variable for each dataset used.

Supplementary File 2 3D projection of the relationship between all disease groups via the overall gene expression of the individual subjects onto an assumed continuum of disease progression in pseudotime. Each dot represents an individual and is color-mapped to the disease group.

Supplementary File 3 Top 10 genes, aka Garnett markers, expressed specifically in each of the groups.

Supplementary File 4 List of the DEGs obtained from all pair-wise comparisons of disease groups and clusters.

Supplementary File 5 List of enriched GO terms obtained from analyses of DEG lists.
