# Supplementary material for: The molecular landscape of sepsis severity in infants: enhanced coagulation, innate immunity, and T cell repression
Source: Front Immunol. 2024 May 16;15:1281111. doi: 10.3389/fimmu.2024.1281111 (PMC11137207; doi:10.3389/fimmu.2024.1281111)

SUPPLEMENTAL INFORMATION

Definition of bacterial sepsis and septic shock.
For dataset GSE64456 bacteremia was defined as the growth of a single pathogen based on blood culture results (25 Bacteremia, 15 Healthy Controls). For the Septic Shock group, the two datasets used established criteria of the International Pediatric Sepsis Consensus Conference (IPSCC) 2005 (see excerpt from Goldstein *et al.*). The two datasets listed below contributed to our Bacteremia group and were clinically assessed in the respective original studies using the following criteria:

1. (GSE25504: 64 sepsis, 71 healthy controls). Positive blood culture and clinical assessment confirmation as defined by respiratory, cardiovascular, and/or metabolic symptoms, temperature instability, feeding intolerance, lethargy/low tone, jaundice, and/or ill appearance/poor color.
2. (GSE69686: 64 sepsis, 85 healthy controls). At the discretion of the physician, diagnosis was established with three prerequisite criteria: i. persistent abnormal clinical examination (see list criteria as per Wynn *et al.*), ii. Positive blood culture, and iii. CRP > 45 mg/L within 8 hours of evaluation.

Excerpt from Goldstein et al. (Pediatr Crit Care Med 2005; 6:2–8) and applicable to datasets GSE26378 and GSE26440.

<u>Sepsis</u>

Sepsis is defined as systemic inflammatory response syndrome (SIRS)[#] in the presence of or as a result of suspected or proven infection*.

[#] <u>SIRS</u>: The presence of **<u>at least two</u>** of the following four criteria, one of which must be abnormal temperature or leukocyte count: core temperature of 38.5°C or 36°C OR leukocyte count elevated or depressed for age (not secondary to chemotherapy-induced leukopenia) or 10% immature neutrophils AND:

- Tachycardia, defined as a mean heart rate 2 SD above normal for age in the absence of external stimulus, chronic drugs, or painful stimuli; or otherwise, unexplained persistent elevation over a 0.5- to 4-hr time period OR for children <1 year old: bradycardia, defined as a mean heart rate <10th percentile for age in the absence of external vagal stimulus, -blocker drugs, or congenital heart disease; or otherwise, unexplained persistent depression over a 0.5-hr time period OR
- Mean respiratory rate 2 SD above normal for age or mechanical ventilation for an acute process not related to underlying neuromuscular disease or the receipt of general anesthesia.

* <u>Infection</u>: A suspected or proven (by positive culture, tissue stain, or polymerase chain reaction test) infection caused by any pathogen OR a clinical syndrome associated with a high probability of infection. Evidence of infection includes positive findings on clinical exam, imaging, or laboratory tests (e.g., white blood cells in a normally sterile body fluid, perforated viscus, and chest radiograph consistent with pneumonia, petechial or purpuric rash, or purpura fulminans).

<u>Septic shock</u>

Septic shock was defined as sepsis and cardiovascular organ dysfunction. Cardiovascular dysfunction is composed of the following observation despite administration of isotonic intravenous fluid bolus >=40 mL/kg in 1 hour:

- Decrease in BP (hypotension) <5th percentile for age or systolic BP <2 SD below normal for age OR
- Need for vasoactive drug to maintain BP in normal range (dopamine>5 g/kg/min or dobutamine, epinephrine, or norepinephrine at any dose) OR
- Two of the following: Unexplained metabolic acidosis: base deficit >5.0 mEq/L, Increased arterial lactate >2 times upper limit of normal, Oliguria: urine output <0.5 mL/kg/hr, Prolonged capillary refill: >5 secs, Core to peripheral temperature gap >3°C.

Discretionary criteria for diagnosis of sepsis as per Wynn et al. and applicable to GSE69686.

General appearance, respiratory signs, need for respiratory support, cardiovascular signs, need for cardiovascular support, neutropenia (ANC <1500), >30% drop in WBC in 24 hours, CRP >45, base deficit, lactate levels, and isolation of any pathogens. Further details can be found in Wynn *et al*. Mol Med. 2015 Jun 2;21(1):496-504.

SUPPLEMENTAL INFORMATION – FIGURES AND TABLES.

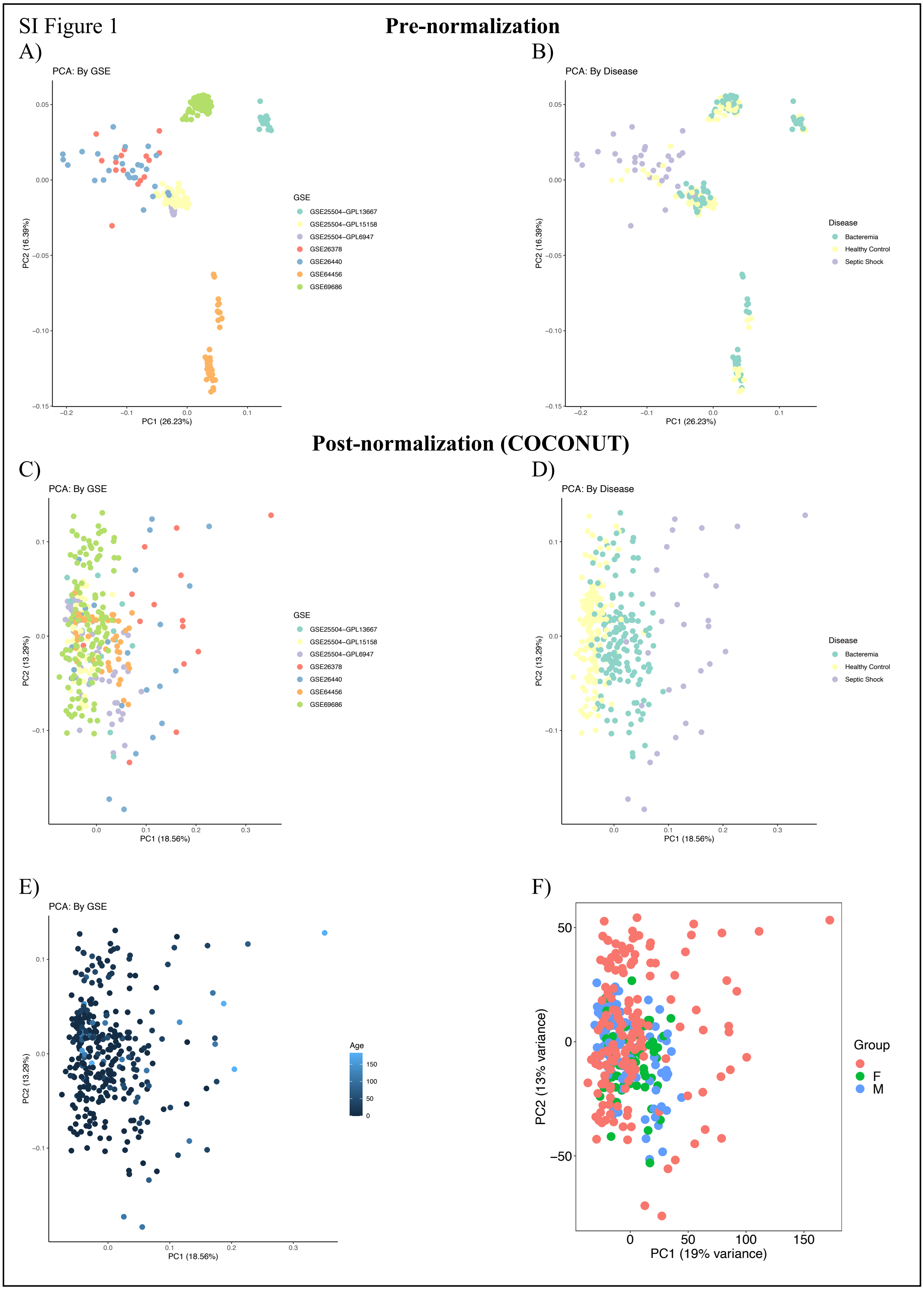

SI Figure 1. Multivariate analyses of the merged dataset pre- and post-COCONUT normalization. A) In the pre-normalization data, gene expression profile is separated by the type of microarray platform and data series. B) Conversely, the pre-normalized data show weak separation based on disease category. C) Post-normalization, we achieved a more uniform distribution of gene expression across series and platform and D) enhanced the separation between disease groups. The global gene expression does not show effects of the E) age and F) sex of the subjects.

SI Figure 2

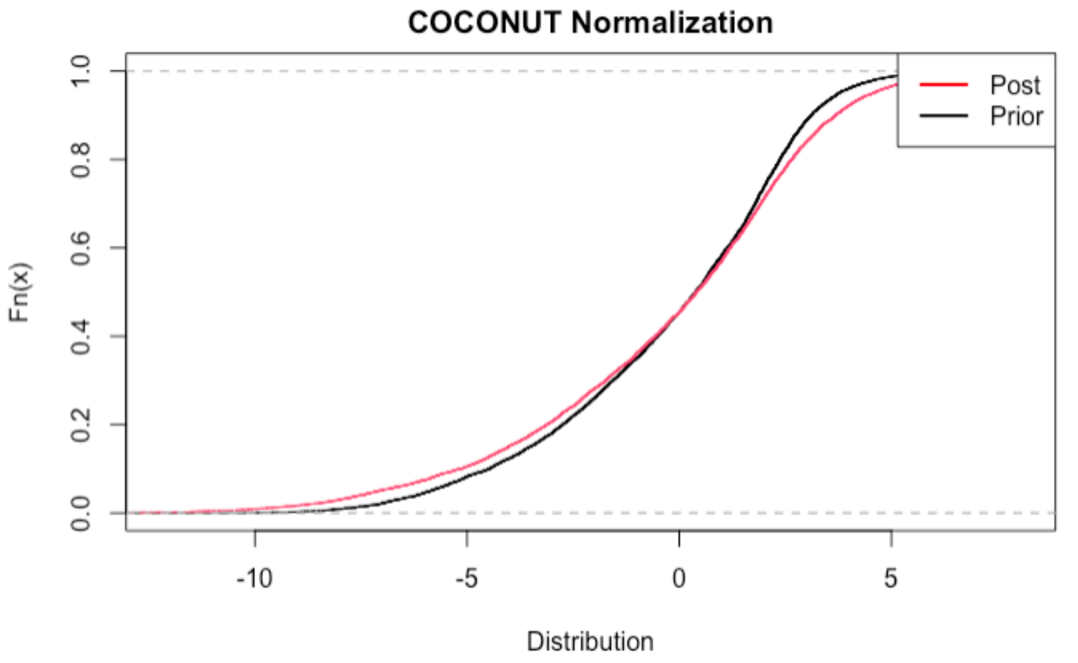

SI Figure 2. The relationship between cases and controls is preserved. Pearson's correlation (cor = 0.982, p-value < 2.2e-16) and Empirical Cumulative Distribution Function were used to assess the prior and post normalized distributions and the relationships between the control and case groups. The y-axis denotes the data percentile and can be interpreted as the probability of an event at the intersection with the x-axis value. The x-axis represents the observed values.

Signature name	Sepsis MetaScore	7-Genes in Neonates	25-Genes in Pediatrics	3-gene signature from pediatrics	SeptiCyte LAB Score	FAIM3:PLAC8 Ratio	6-Genes in Geriatric
**Short name**	**SMS**	**NS**	**PD25**	**PD3**	**SLS**	**RG**	**GD**
**Title of reference article**	Validation of the Sepsis MetaScore for diagnosis of neonatal sepsis (PMID: 28419265)	Potential genes and pathways of neonatal sepsis based on functional gene set enrichment analysis (PMID: 30154914)	Transcriptomic meta-analysis reveals up-regulation of gene expression functional in osteoclast differentiation in human septic shock (PMID: 28199355)	Reverse Engineering of the Pediatric Sepsis Regulatory Network and Identification of Master Regulators	A Molecular Host Response Assay to Discriminate Between Sepsis and Infection-Negative Systemic Inflammation in Critically Ill Patients: Discovery and Validation in Independent Cohorts (PMID: 26645559)	A molecular biomarker to diagnose community-acquired pneumonia on intensive care unit admission (PMID: 26121490)	Distinguishing septic shock from non-septic shock in postsurgical patients using gene expression (PMID: 34144116)
**Year**	2018	2018	2017	2021	2015	2015	2021
**Dataset Used**	GSE25504, GSE69686, E-MTAB-4785	GSE11755	GSE4607, GSE8121, GSE9692, GSE13904, GSE26378, GSE26440	GSE13904, GSE4607, GSE26378, GSE56649, GSE21942	NCT01905033	NCT01905033	GSE131761
**Age group**	Up to 3 months	Unknown	< 10 years	mix of < 10 years and adults	>18 years	>18 years	> 65 years
**Gene list**	CEACAM1, ZDHHC19, C9ORF95, GNA15, BATF, C3AR1, KIAA1370, TGFBI, MTCH1, RPGRIP1, HLA-DPB1	PIK3CA, TGFBR2, CDKN1B, KRAS, E2F3, TRAF6, CHUK	JUNB, IL1B, PLCG2, SYK, NFKBIA, MAPK1, GAB2, NCF2, IFNGR1, LILRB2, IFNAR2, TYROBP, IFNAR1, PIK3CG, FCGR2A, LILRA3, SPI1, NCF4, IFNGR2, MAPK3, SIRPA, OSCAR, LILRA2, FCGR1A, LILRA6	TRIM25, RFX2, MEF2A	CEACAM4, LAMP1, PLA2G7, PLAC8	FAIM3, PLAC8	IL1R2 , LCN2 , LTF , MMP8 , OLFM4

SI Table 1. Assessment of selected public sepsis gene signatures. Description of source articles, year published, related dataset ID number, age of the subject evaluated, and gene list that composed each signature.

SI Figure 3

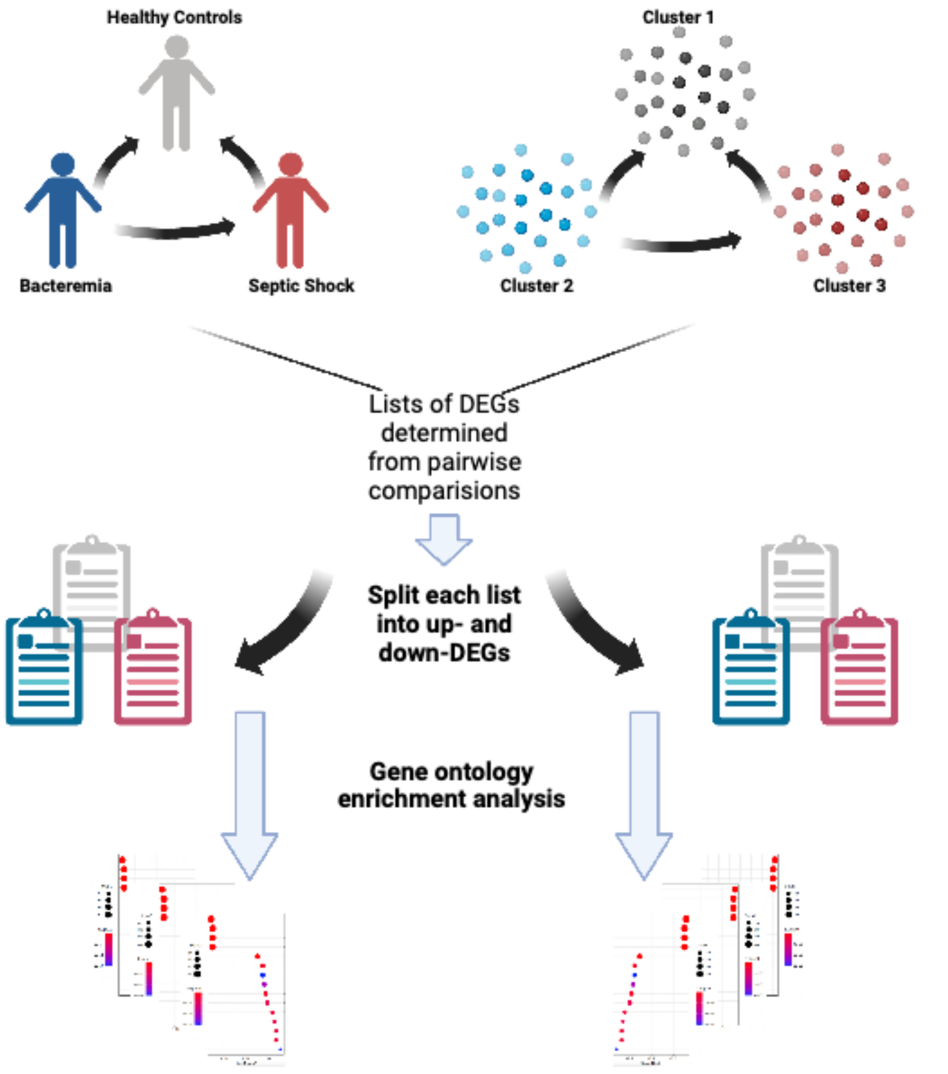

SI Figure 3. Workflow of the comparative analysis approach taken to delineate markers of sepsis severity.

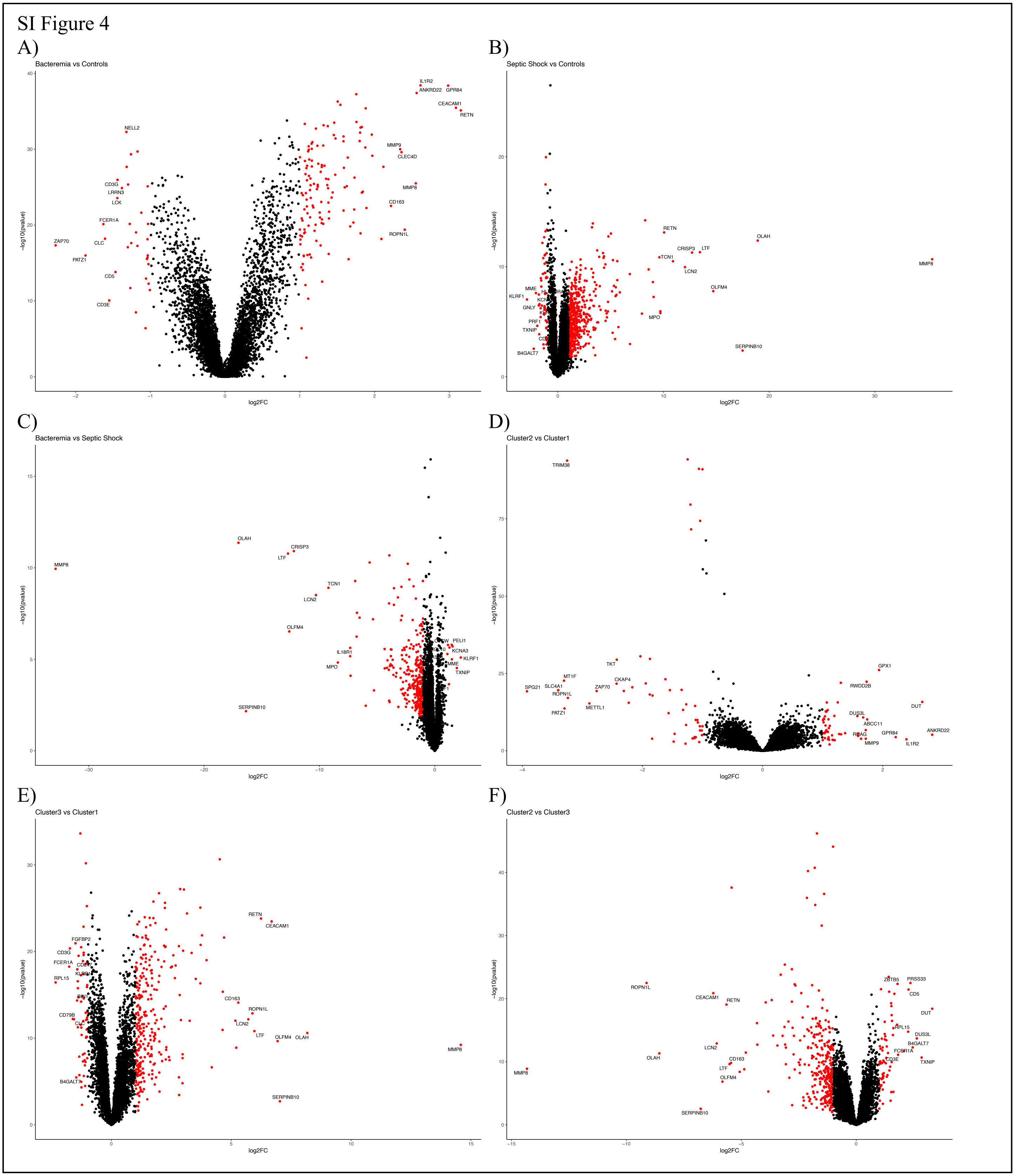

SI Figure 4. Differential gene expression analysis comparing disease groups and pseudotime clusters. A-B) Volcano plots representation of DEGs in each group against Healthy Controls and C) for Bacteremia against Septic Shock. D-E) Volcano plots representation of DEGs in each cluster against #1 and F) for Cluster 2 against Cluster 3. Analyses was performed using two-sided Wilcox Rank test. Significance of the DEGs were denoted by the following thresholds: $|log_2FC| > 1$ and p.adjust < 0.05.

Common between Bacteremia and Septic Shock

UP	Bacteremia	Septic Shock	DOWN	Bacteremia	Septic Shock
RETN	3.15	10.06	NOG	-1.12	-1.25
CEACAM1	3.09	9.61	ITM2A	-1.17	-1.27
GPR84	2.98	5.63	KLRB1	-1.19	-1.63
IL1R2	2.61	8.26	PRSS33	-1.19	-1.21
ANKRD22	2.56	5.13	RPL15	-1.27	-1.14
MMP8	2.55	35.42	LRRN3	-1.38	-1.07
ROPN1L	2.40	9.70	CD3G	-1.44	-1.25
CLEC4D	2.36	3.32	CD3E	-1.55	-1.76
MMP9	2.34	5.31	CLC	-1.61	-1.06
CD163	2.22	9.00	FCER1A	-1.63	-1.56

Common between clusters 2 and 3

UP	Cluster 2	Cluster 3	DOWN	Cluster 2	Cluster 3
ANKRD22	2.82	3.78	CD5	1.08	-1.19
IL1R2	2.39	4.70	SPTA1	1.06	1.69
GPR84	2.22	4.52	TMCC2	1.04	1.81
RWDD2B	1.73	1.11	CLIC2	1.04	1.76
MMP9	1.72	3.72	HMBS	1.03	1.04
RHAG	1.72	2.29	ALAS2	1.02	1.11
TNFAIP6	1.64	1.52	PLSCR1	1.00	1.68
TXNIP	1.59	-1.25	CLC	-1.84	-1.56
ACSL1	1.31	1.18	CKAP4	-2.43	2.99
HBZ	1.27	1.71	ROPN1L	-3.24	5.88

SI Table 2. Varying degree of modulation for the commonly perturbed genes among the disease groups against Healthy Controls (top panel) and the pseudotime clusters against Cluster 1. Shown is the log_2FC values of the top 10 up- and down-DEGs which are ranked based on the Bacteremia group (out of 125 common DEGs) or Cluster 2 (out of 29 common DEGs).

Unique DEGs obtained via disease group comparisons.

↑ Unique up-regulated ↑ (log2FC)		↓ Unique down-regulated ↓ (log2FC)	
**Bacteremia**	**Septic shock**	**Bacteremia**	**Septic shock**
TNFAIP6 (1.38)	LTF (13.4)	FAM102A (-1.2)	CTSW (-1.5)
S100A8 (1.37)	CRISP3 (12.6)	BIN1 (-1.2)	MS4A1 (-1.6)
NFKBIA (1.31)	MS4A3 (7.95)	CD247 (-1.2)	PRF1 (-1.6)
KCNJ2 (1.29)	CTSG (6.83)	DENND2D (-1.3)	KCNA3 (-1.7)
CASP5 (1.27)	TUBB2A (5.41)	OCIAD2 (-1.3)	HLA-DRA (-1.7)
CCR1 (1.18)	STOM (4.78)	NELL2 (-1.3)	GNLY (-1.8)
SPI1 (1.16)	RNASE3 (4.74)	LCK (-1.4)	TXNIP (-1.9)
SLC1A3 (1.13)	VSIG4 (4.73)	CD5 (-1.4)	MME (-2.0)
HCK (1.12)	HGF (4.64)	PATZ1 (-1.8)	B4GALT7 (-2.2)
TYROBP (1.10)	RHAG (4.52)	ZAP70 (-2.2)	KLRF1 (-2.9)

Unique DEGs obtained via pseudotime cluster comparisons.

↑ Unique up-regulated ↑ (log2FC)		↓ Unique down-regulated ↓ (log2FC)	
**Cluster 2**	**Cluster 3**	**Cluster 2**	**Cluster 3**
DUT (2.65)	MMP8 (14.5)	FZD2 (-2.2)	OCIAD2 (-1.4)
GPX1 (1.93)	OLAH (8.17)	MNT (-2.3)	KLRB1 (-1.4)
ABCC11 (1.74)	SERPINB10 (7.02)	TKT (-2.4)	CD247 (-1.4)
DUS3L (1.67)	CEACAM1 (6.68)	ZAP70 (-2.7)	BIN1 (-1.4)
RSAD2 (1.58)	RETN (6.24)	METTL1 (-2.8)	B4GALT7 (-1.4)
DLK2 (1.57)	LTF (5.96)	TRIM38 (-3.2)	FGFBP2 (-1.4)
EPB42 (1.37)	LCN2 (5.71)	PATZ1 (-3.2)	CD79B (-1.6)
GIGYF1 (1.30)	CD163 (5.29)	MT1F (-3.3)	CD3G (-1.7)
NFS1 (1.11)	CRISP3 (5.20)	SLC4A1 (-3.4)	FCER1A (-1.7)
RAP1GAP (1.10)	TCN1 (5.17)	SPG21 (-3.9)	RPL15 (-2.3)

SI Table 3. Top 10 unique up- and down-regulated genes, by fold-changes, in the Bacteremia and Septic Shock groups when compared with Healthy Controls (top panel) and in pseudotime clusters 2 and 3 when compared to cluster 1 (bottom panel). Shown in parentheses are $\log_2$FC values.

SI Figure 5

A)

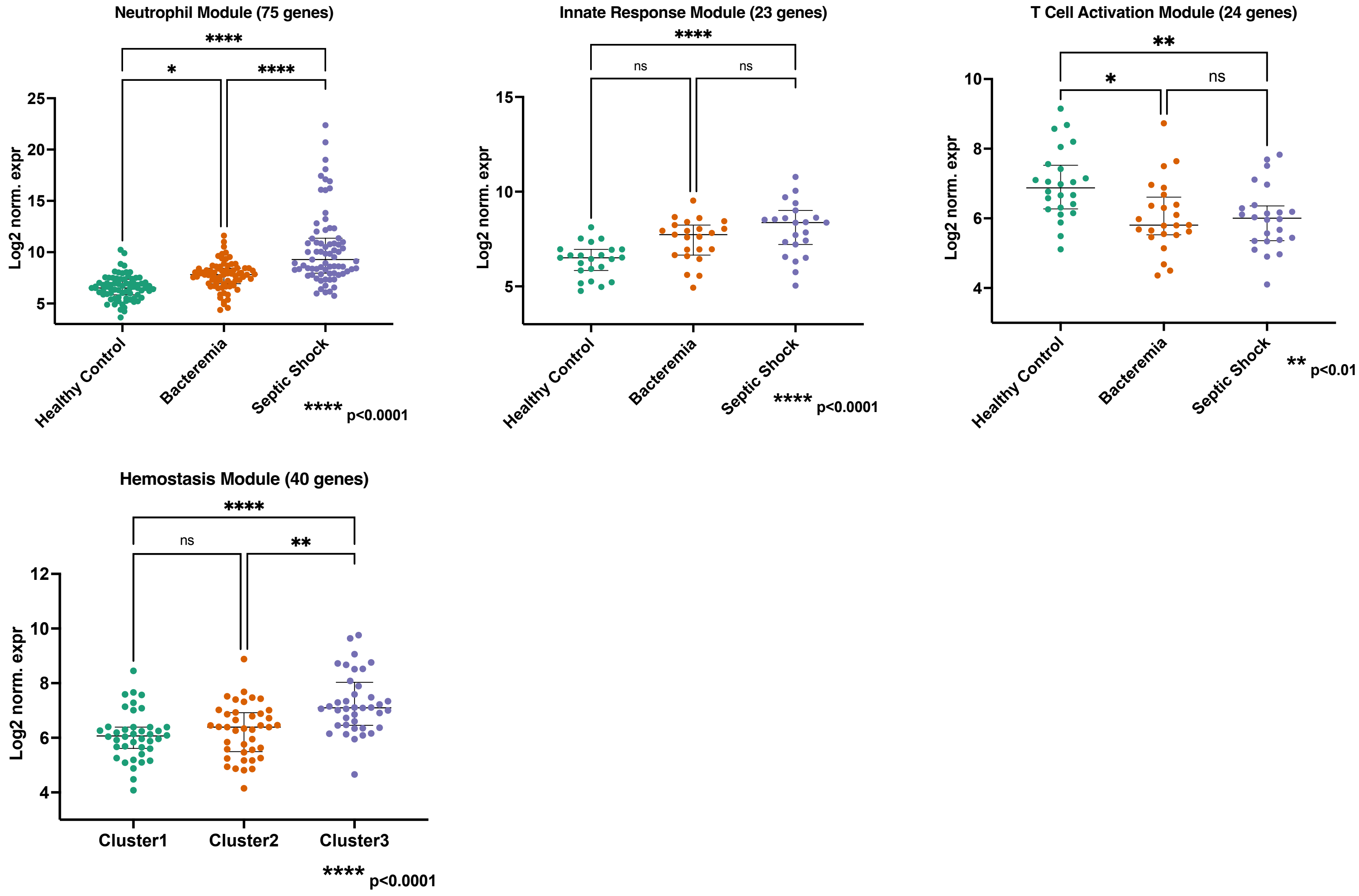

SI Figure 5. Gene expression pattern of important modules. Genes belonging to neutrophils (GO:0042119, GO:0043312, GO:0002283, GO:0002446), innate immune responses (GO:0050727, GO:0045088, GO:0002269), T cell activation/receptor (GO:0042110, GO:0030098, GO:0050852, GO:0050863, GO:0050870, GO:0042129, GO:0046651, GO:0002285), and hemostasis (GO:0007596, GO:0007599, GO:0050817, GO:0002576) related processes were independently combined to form modules of 75, 23, 24, and 40 genes, respectively. The $\log_2$ normalized expression values of the genes in each module were plotted for each disease group. Kruskal-Wallis with Dunn's multiple comparisons test was used to assess significant difference between group as indicated.

SI Figure 6

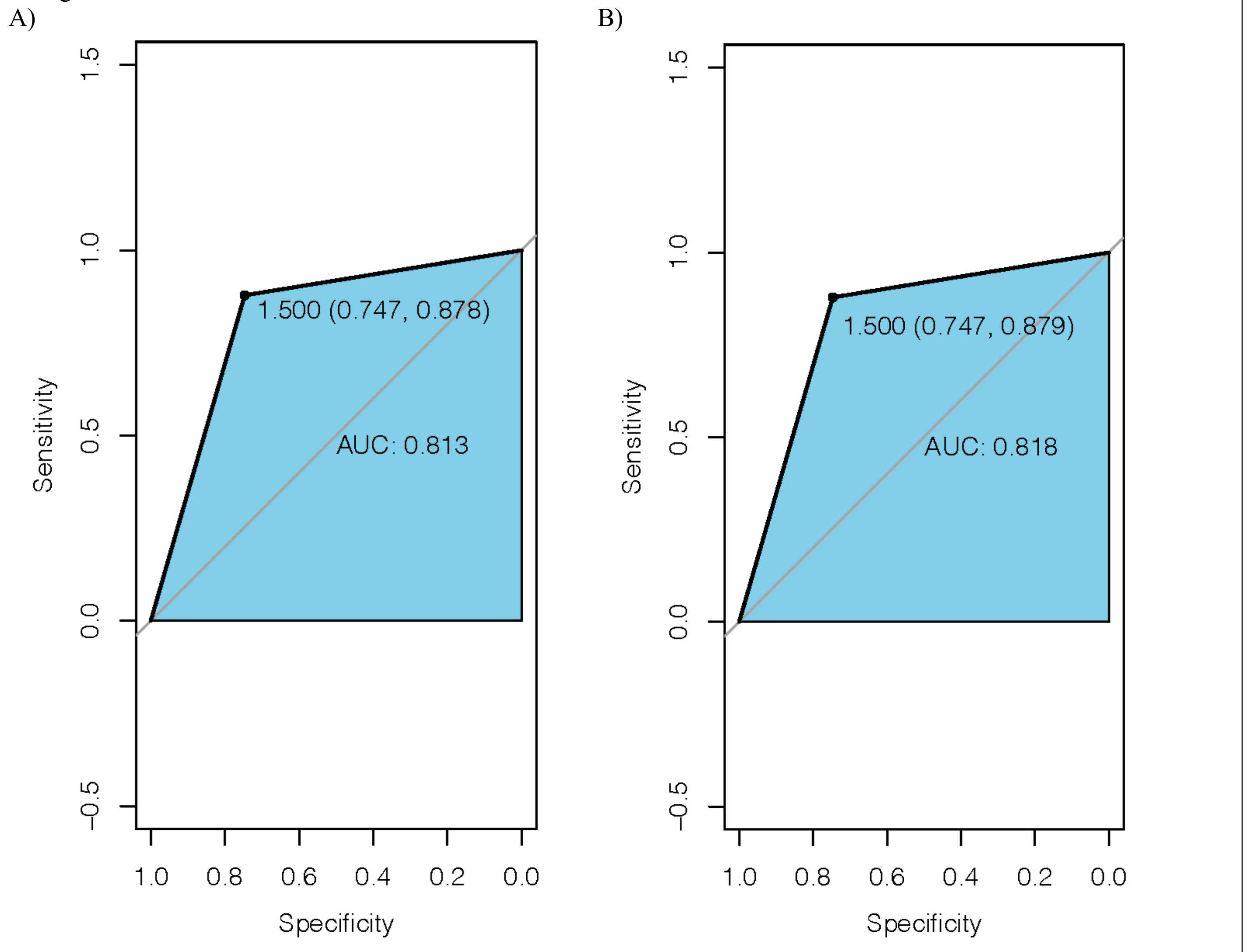

SI Figure 6. ROC curve analysis derived from A) The combination of hemostasis, neutrophils, innate immune responses, and T cell activation modules and B) Garnet marker signature.

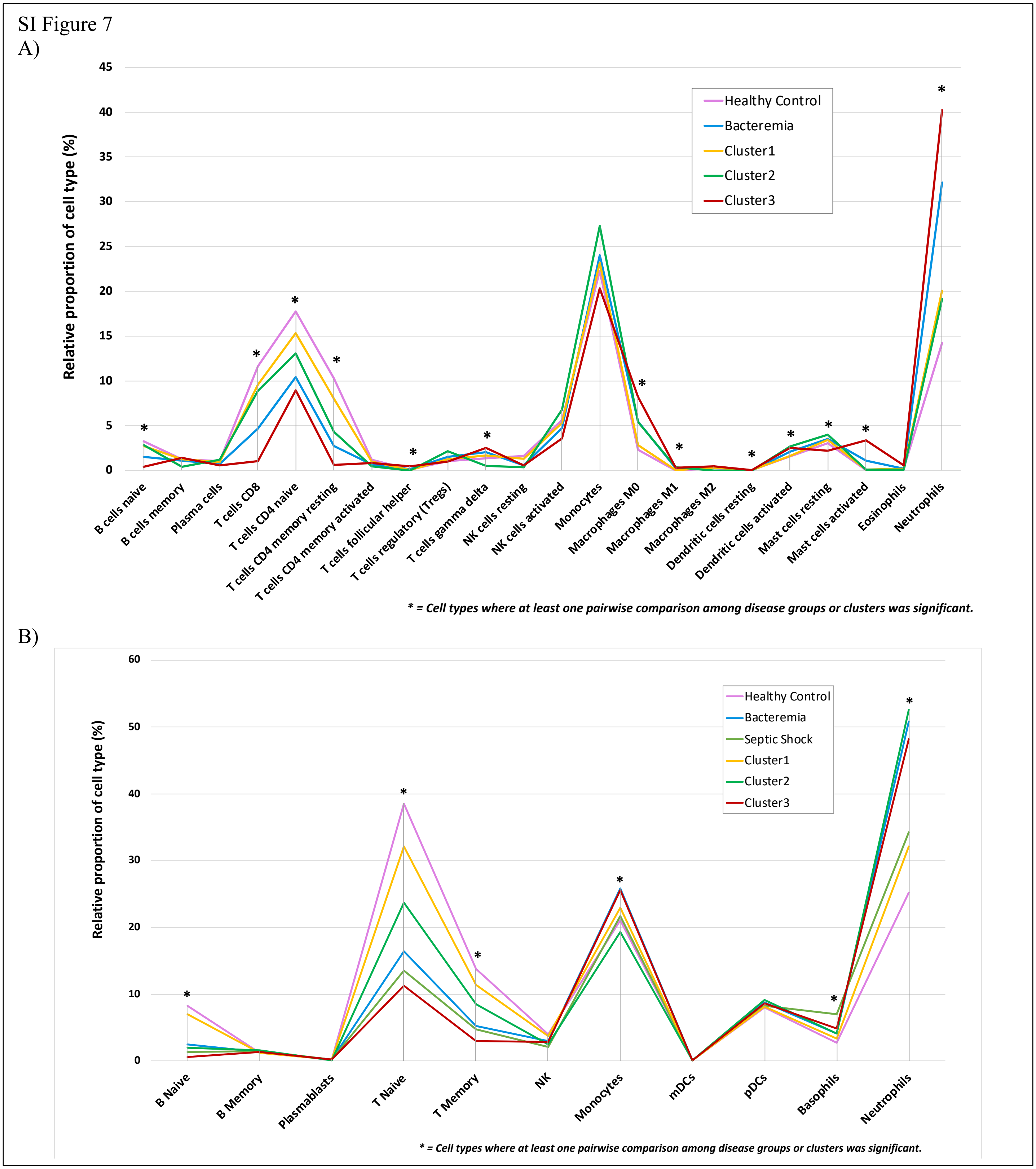

SI Figure 7. Summary of the immune cell deconvolution. Deconvolution from bulk RNA expression data following the approach by A) CIBERSORT using the LM22 single-cell reference dataset and B) ABsolute Immune Signal (ABIS) deconvolution (available at https: //giannimonaco.shinyapps.io/ABIS/). Both results are expressed as the relative proportion of cell type per sample (such that the total of all calculated proportions adds up to 100%). Groups are labelled according to the inset and the asterisk denotes where at least one pair-wise comparison among groups reached significance.

SI Figure 8

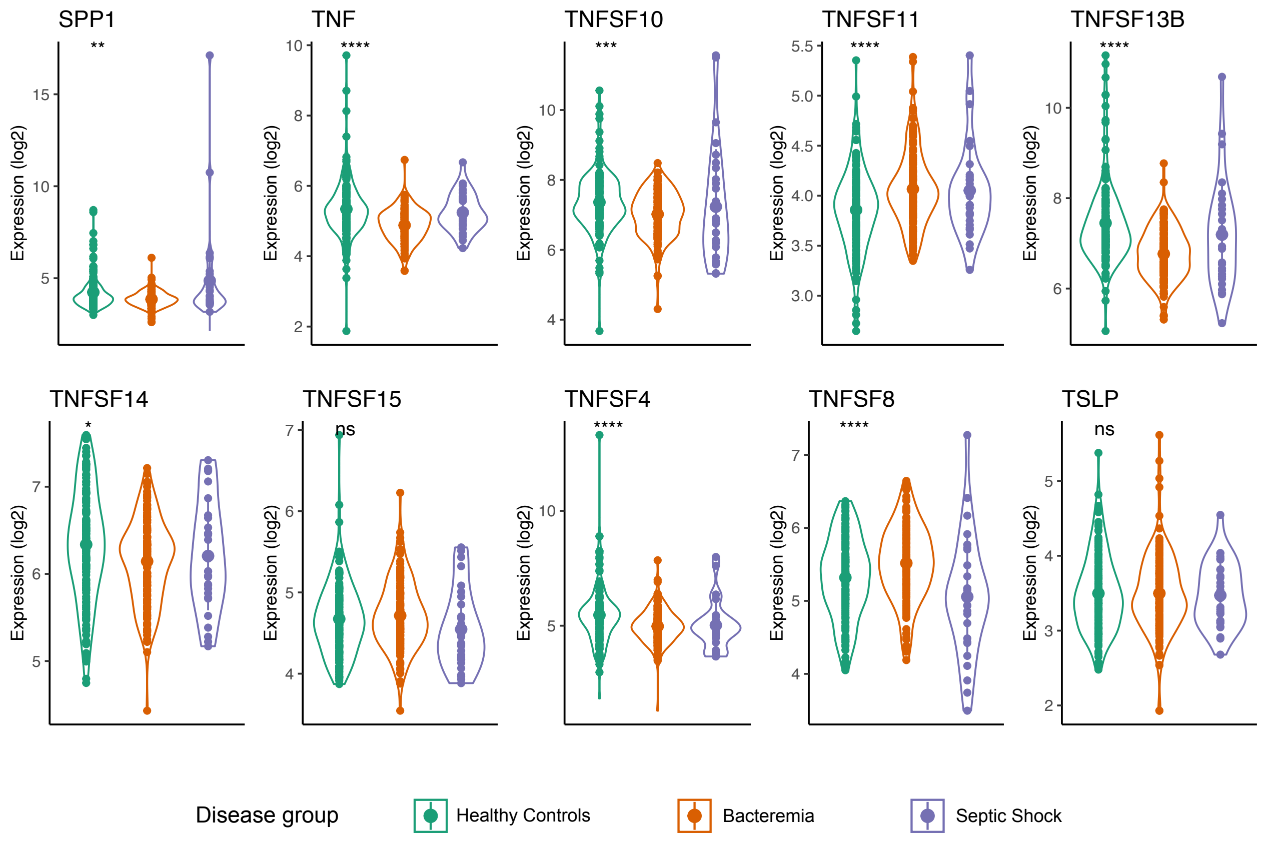

SI Figure 8. Profiles of cytokine, including chemokine, coding genes identified among our co-normalized genes. Each dot represents the log2 normalized expression values. The significance of differences between groups was assessed using the Kruskal-Wallis test with Dunn's multiple comparisons correction.

SI Figure 8 con't

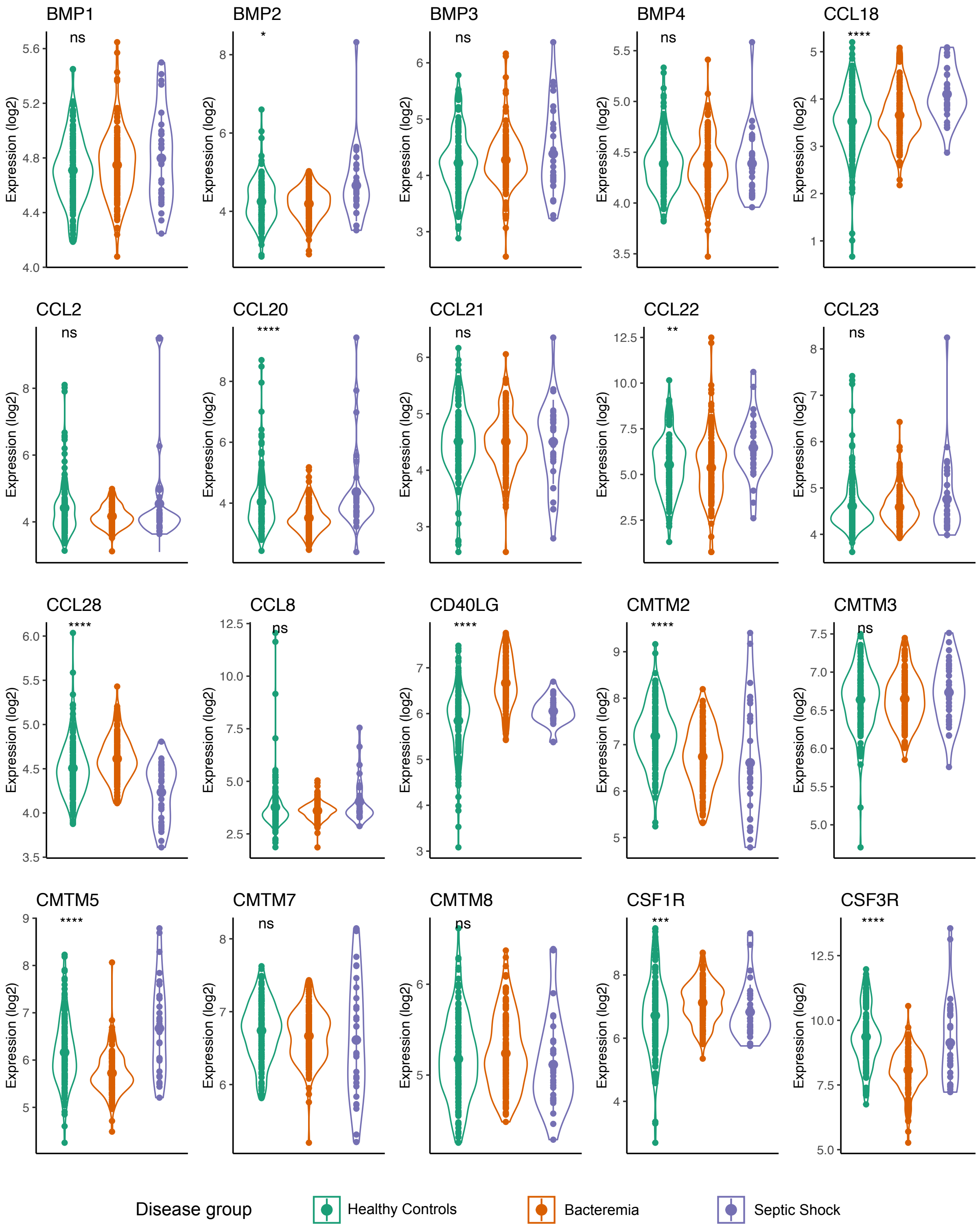

SI Figure 8 con't

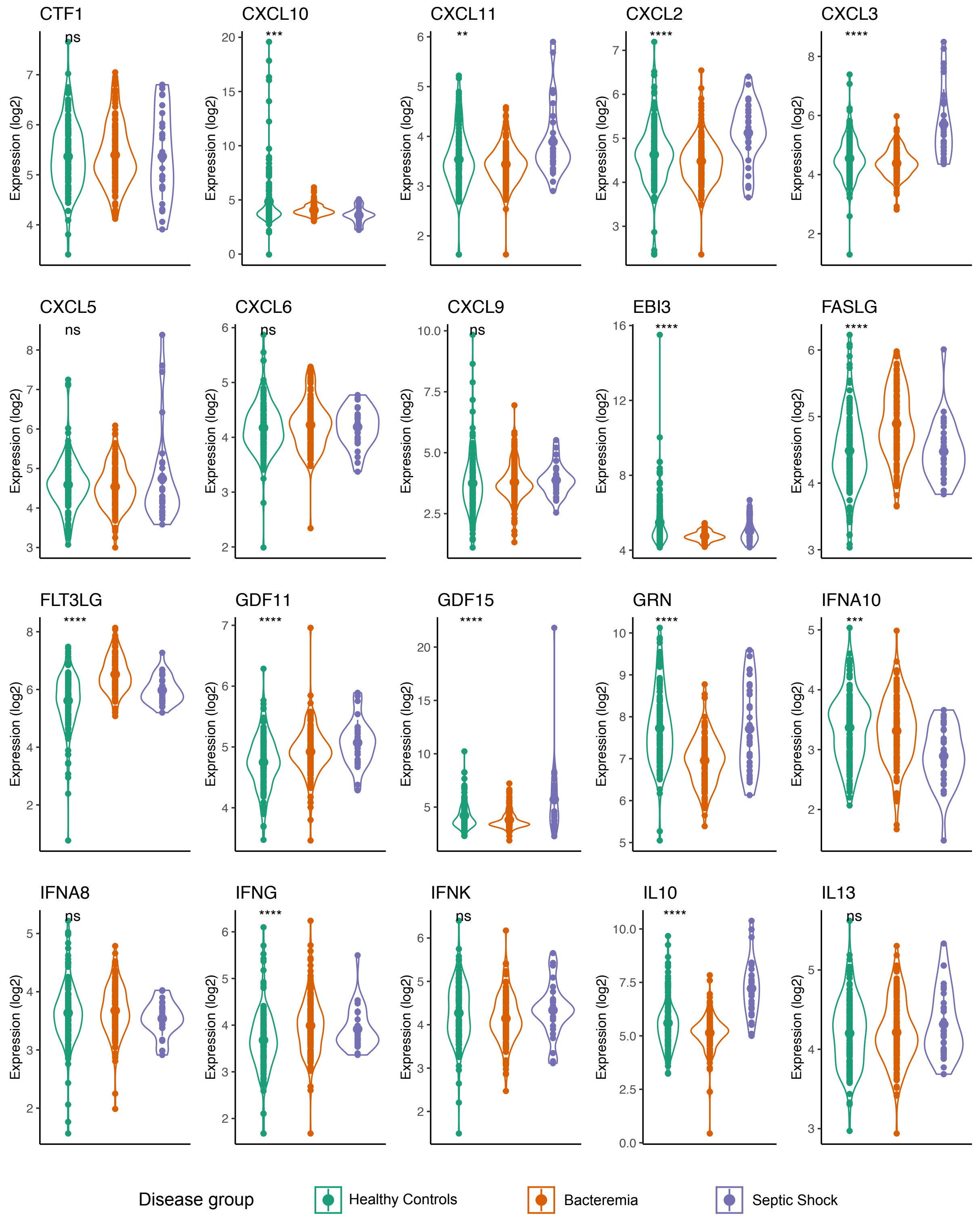

SI Figure 8 con't

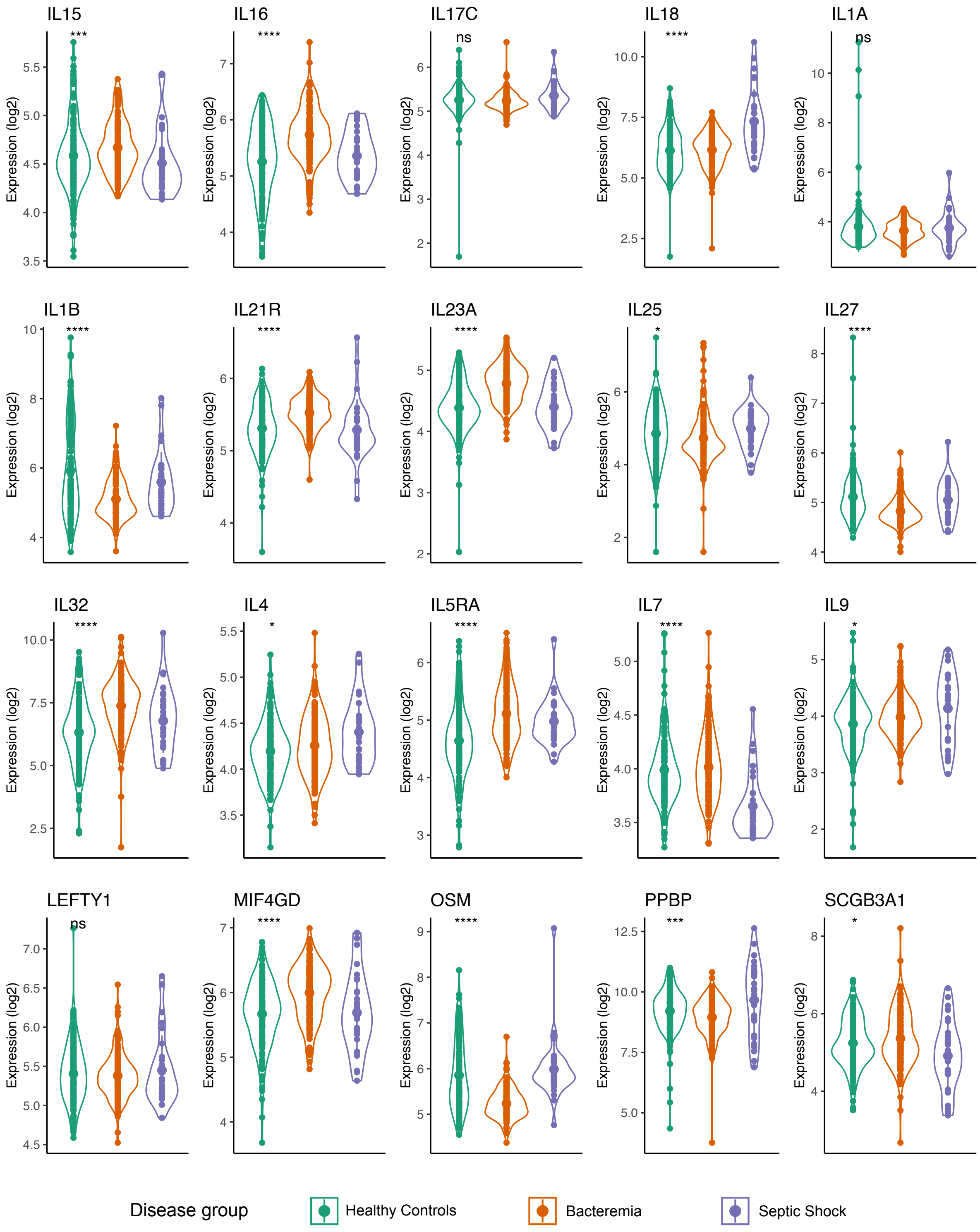

SI Figure 8 con't

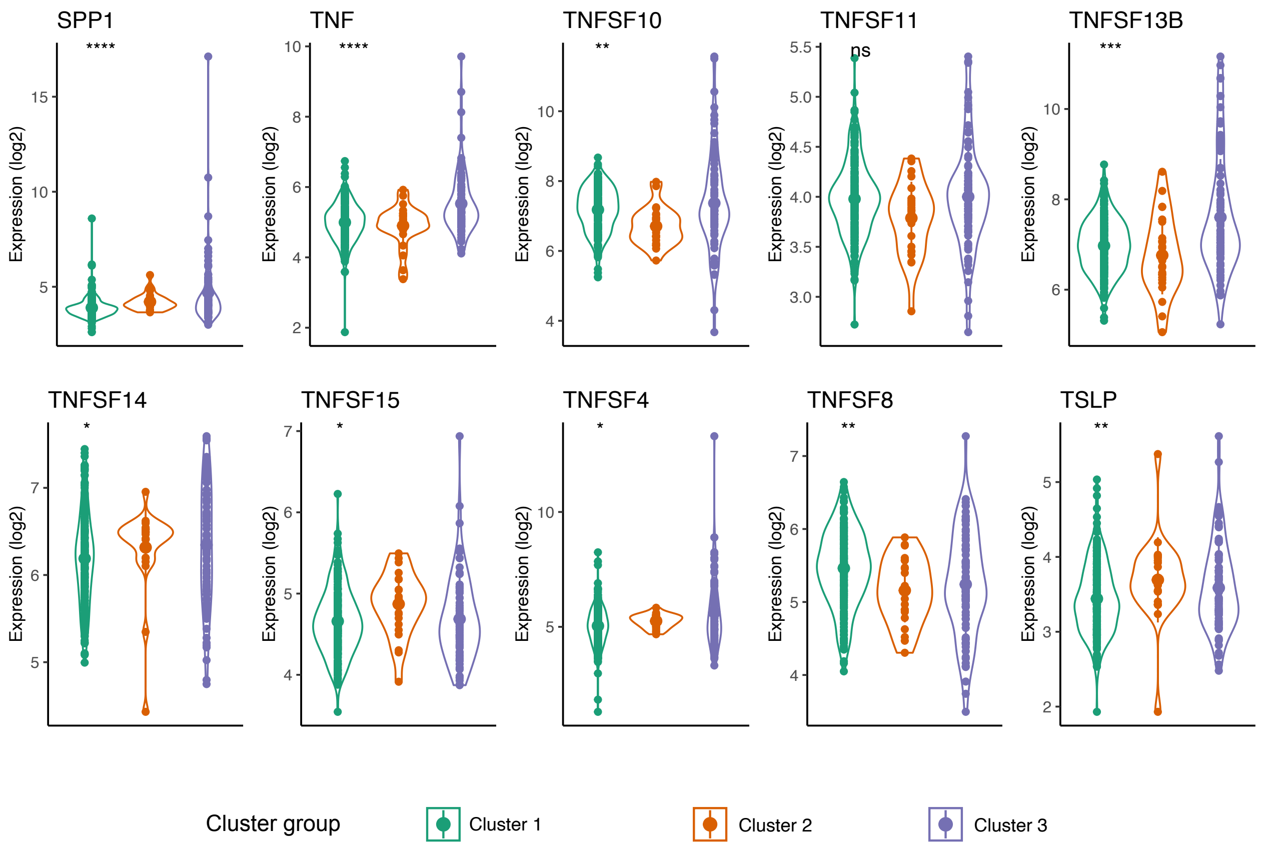

SI Figure 8 con't

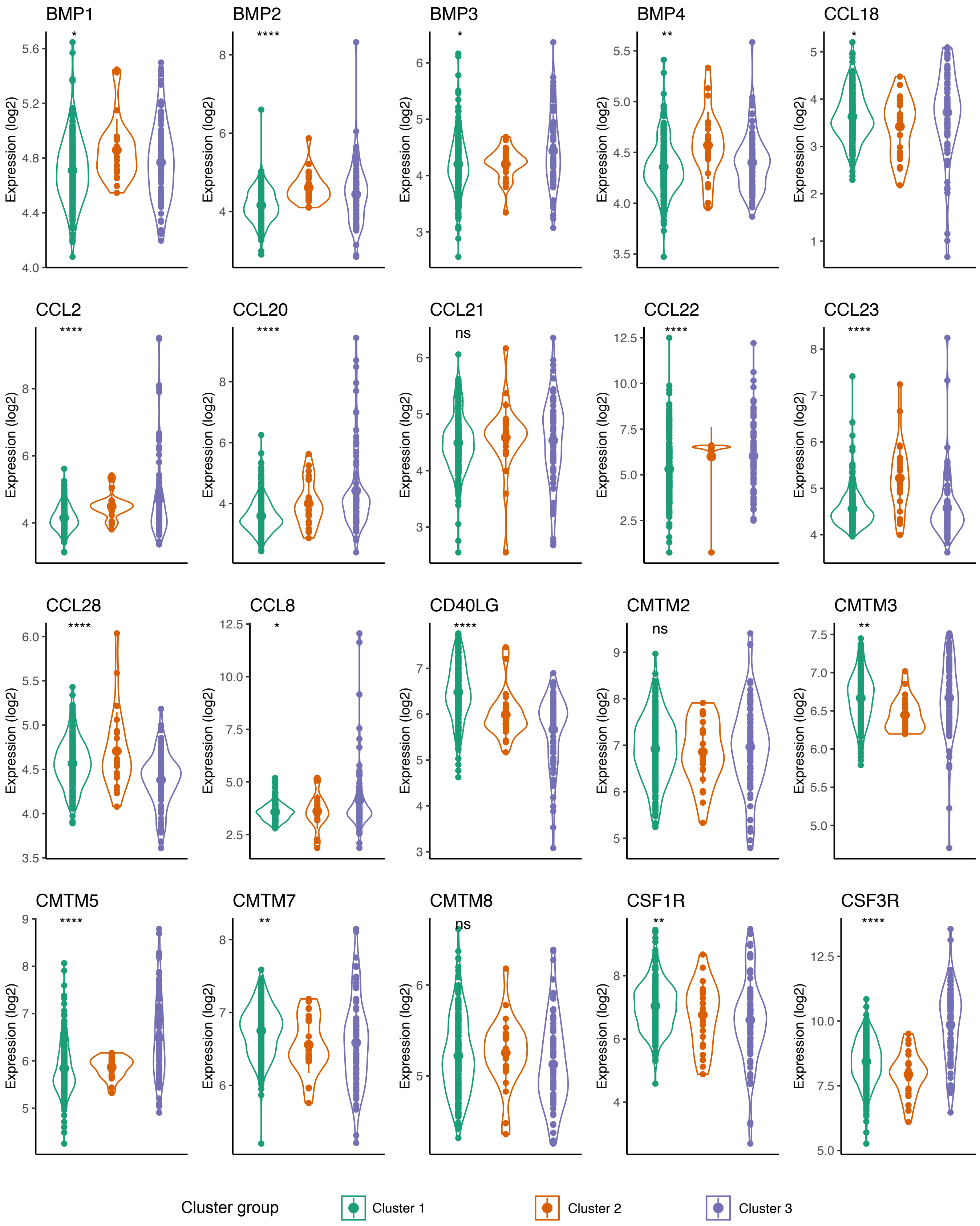

SI Figure 8 con't

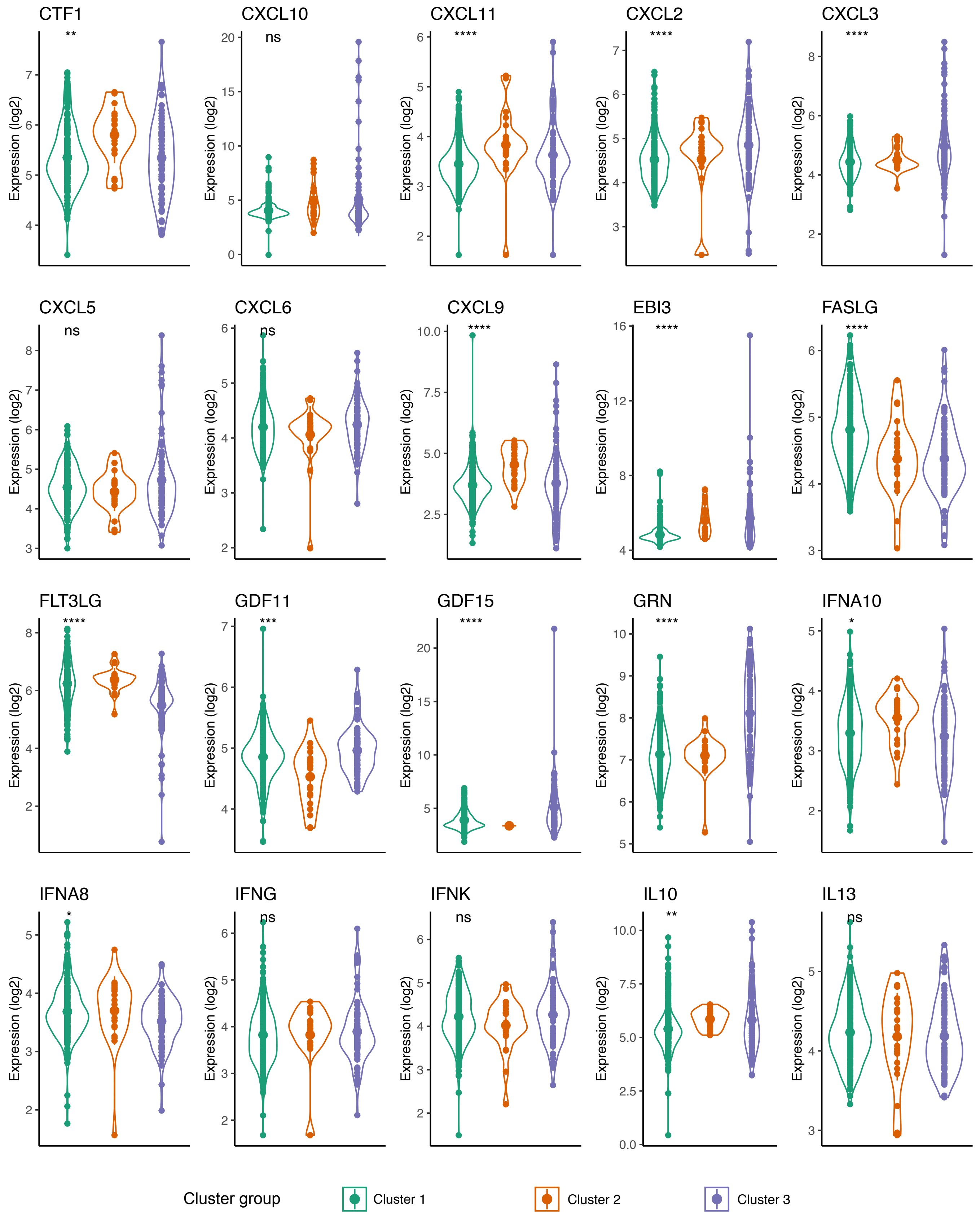

SI Figure 8 con’t

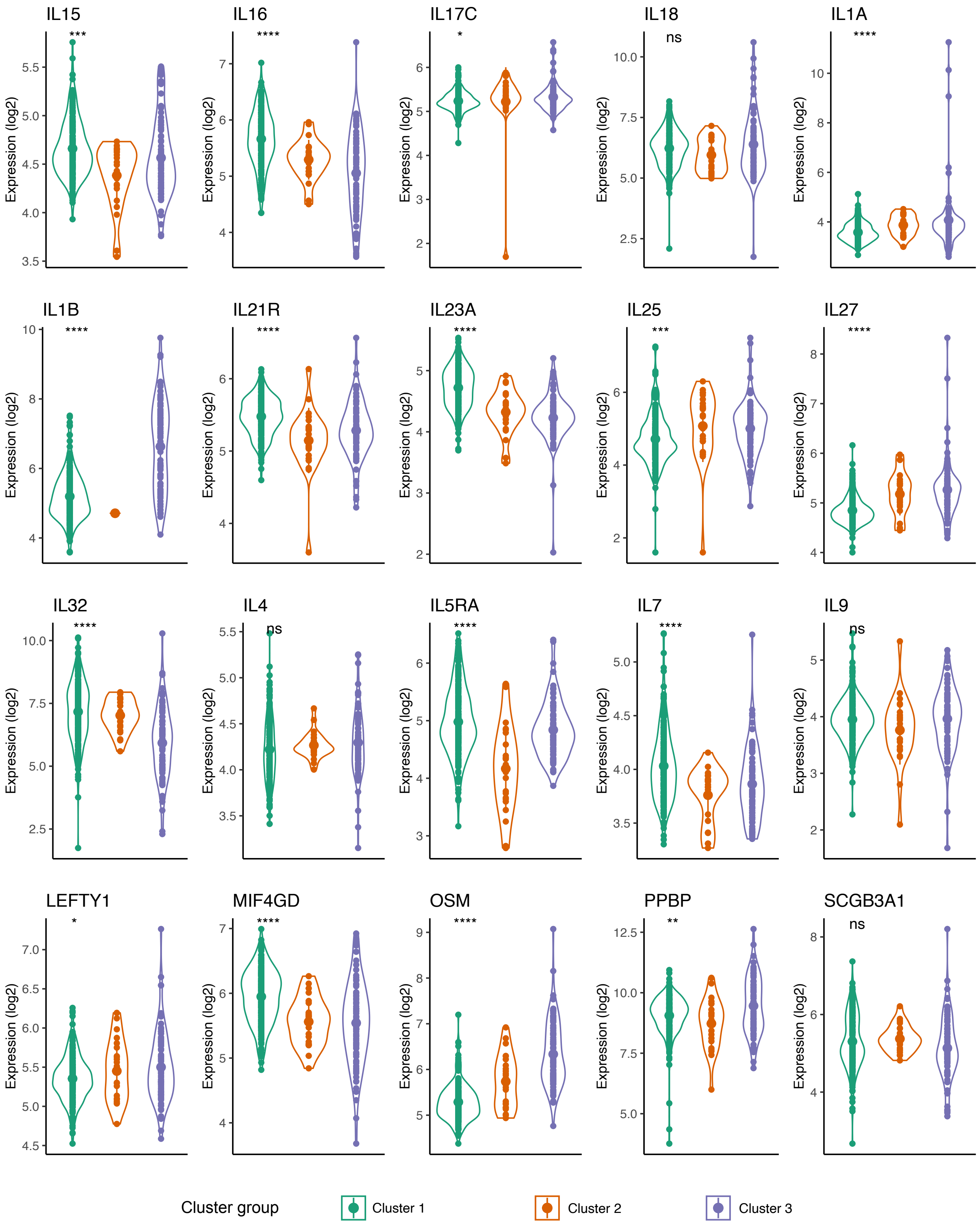

Supplement: Supplementary Figure 1 — Multivariate analyses of the merged dataset pre- and post-COCONUT normalization. (A) In the pre-normalization data, gene expression profiles are separated by the type of microarray platform and data series. (B) Conversely, the pre-normalized data show weak separation based on disease category. (C) Post-normalization, we achieved a more uniform distribution of gene expression across series and platforms and (D) enhanced the separation between disease groups. The global gene expression does not show effects of the (E) age and (F) sex of the subjects. [file DataSheet_1.pdf]
